# Supplementary material for: Data-driven protease engineering by DNA-recording and epistasis-aware machine learning
Source: Nat Commun. 2025 Jul 1;16:5466. doi: 10.1038/s41467-025-60622-7 (PMC12217912; doi:10.1038/s41467-025-60622-7)
Supplement: Supplementary file 1 — Supplementary Information [file 41467_2025_60622_MOESM1_ESM.pdf]

Supplementary Information for:

Data-driven Protease Engineering by DNA-Recording and Epistasis-aware Machine Learning

Lukas Huber[1,†], Tim Kucera[1,2,3,†], Simon Höllerer[1], Karsten Borgwardt[1,2,3]*, Sven Panke[1]*, Markus Jeschek[1,4,5]*

[1]Department of Biosystems Science and Engineering, ETH Zurich, CH-4058 Basel, Switzerland.

[2]Swiss Institute of Bioinformatics, CH-4058 Basel, Switzerland.

[3]Max Planck Institute of Biochemistry, D-82152 Martinsried, Germany.

[4]Synthetic Microbiology, University of Regensburg, D-93053 Regensburg, Germany.

[5]Laboratory of Synthetic and Applied Microbiology, SB ISIC & SV IBI, École Polytechnique Fédérale de Lausanne (EPFL), CH-1015 Lausanne, Switzerland.

[†]These authors contributed equally

*Correspondence: markus.jeschek@epfl.ch, sven.panke@bsse.ethz.ch, borgwardt@biochem.mpg.de

Supplementary information

Supplementary figures

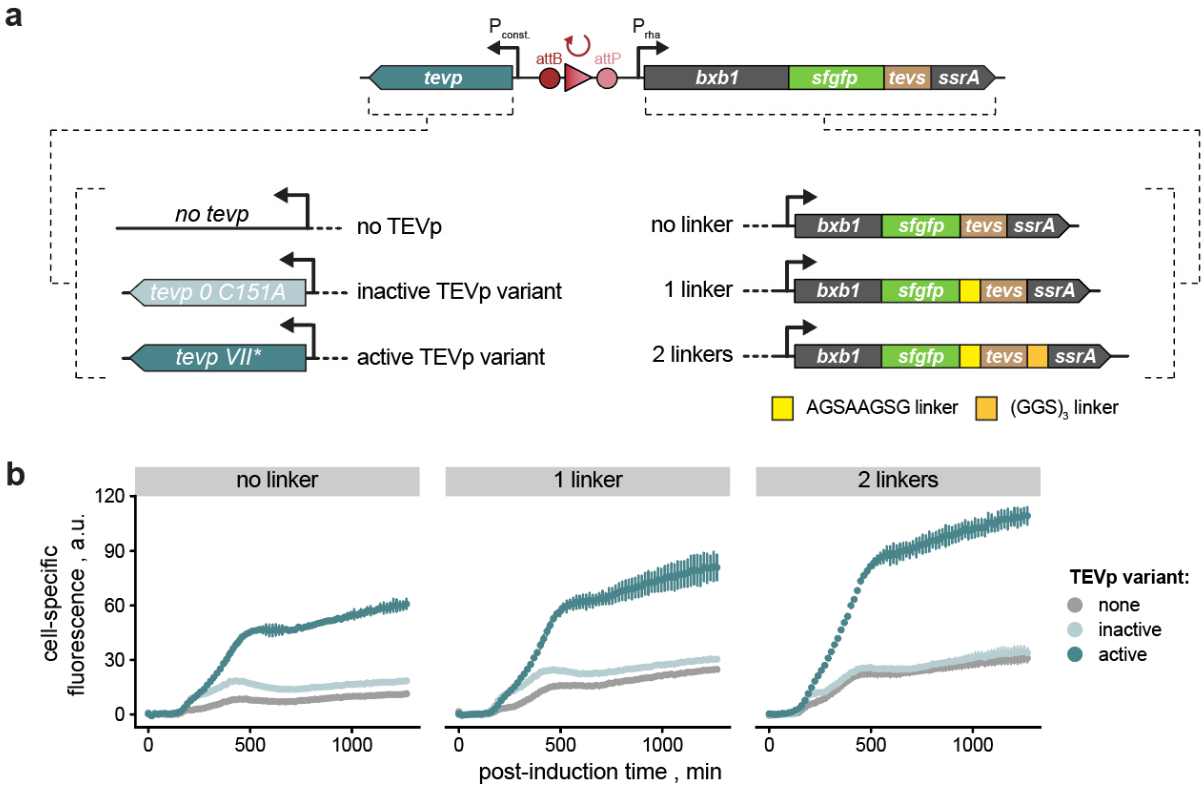

Fig. S1. Investigation of TEVp-dependent stabilization of Bxb1-sfGFP fusions with different linkers flanking TEVs. (**a**) To assess whether TEVp activity leads to an increase in cellular recombinase levels, Bxb1 was translationally fused to sfGFP followed by TEVs and the degradation tag SsrA to facilitate fluorescence measurements indicative of Bxb1 levels. Different versions of the recombinase fusion featuring no, one or two linker(s) adjacent to TEVs were investigated in the absence or presence of active and catalytically inactive TEVp variants. The underlying hypothesis was that an addition of linkers could enhance the accessibility of the substrate for TEVp and thus increase the efficiency of proteolysis leading to faster stabilization of the Bxb1 reporter. TEVp variants and designations are derived from a previous study in our laboratory[1]. Note that *bxb1* is controlled by an RBS of intermediate predicted strength (rel. strength of 0.41[2]). (**b**) Microtiter plate cultivation of *E. coli* strains bearing the different constructs from (a). OD_{600}-normalized fluorescence of Bxb1-sfGFP levels in displayed as mean of n=3 different culture wells with standard deviation. Source data are provided as a Source Data file.

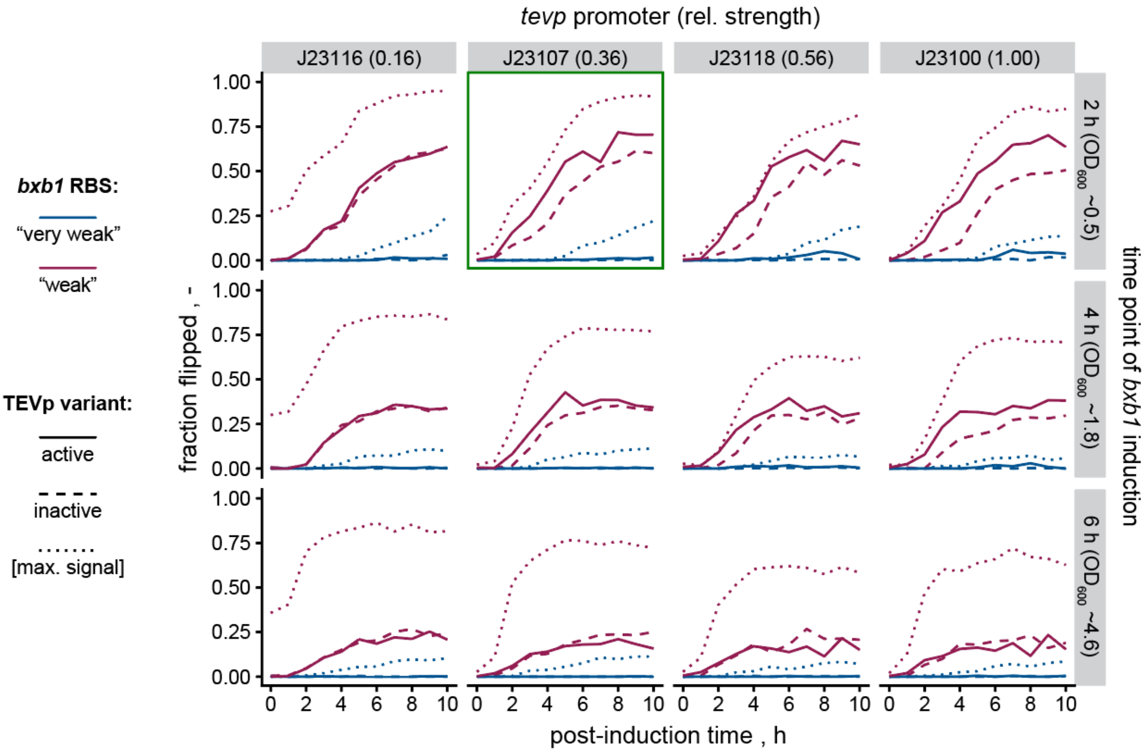

Fig. S2. Fine tuning of DNA recorder by RBS and promoter engineering. For *bxb1,* two RBSs with relative predicted strengths of 0.06 ("very weak") and 0.21 ("weak")[2] were tested. Note that stronger RBSs led to strong leaky recombination and were therefore excluded from the optimization. For *tevp*, four different constitutive promoters from the Anderson collection[3] were tested with numbers in parentheses indicating relative promoter strength. TEVp 0 and TEVp 0 C151A were used as active and inactive TEVp variants, respectively[1]. To test for maximum possible recombination (i.e. max. signal), a construct with TEVp 0 C151A and an SsrA-less Bxb1 fusion was used. Furthermore, the induction time point of *bxb1* after inoculation was varied. As optimal setup (green box), the combination of the weak RBS with promoter J23107 as well as induction at 2h after inoculation was selected since this provides a good trade-off between a good signal over background (i.e. delta between active and inactive TEVp variant) while leaving sufficient dynamic range to resolve proteolytic activity higher than the active TEVp variant (i.e. delta between max. signal and active TEVp variant). Source data are provided as a Source Data file.

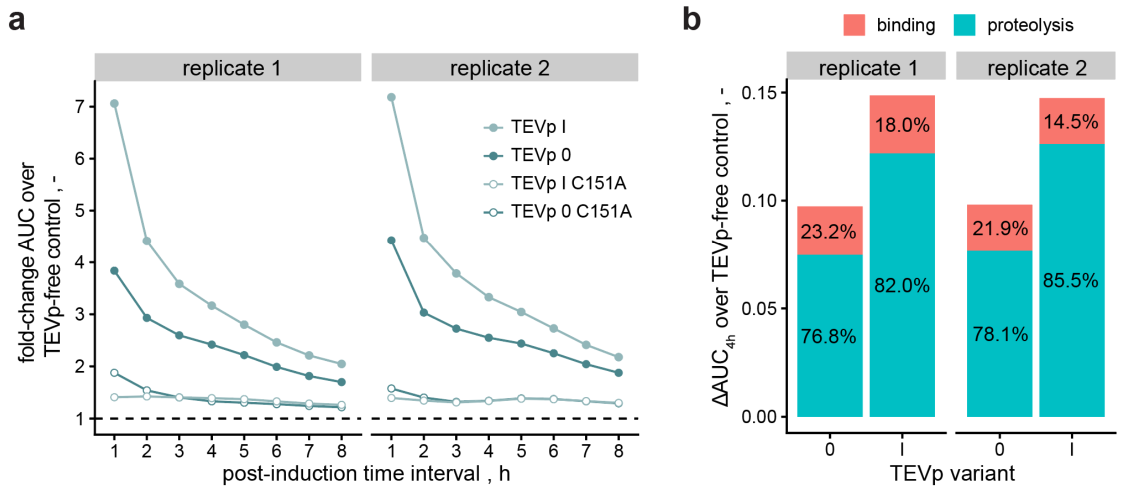

Fig. S3. Investigation of the effect of TEVp binding and proteolysis on DNA recorder activity. (**a**) Impact of the expression of different TEVp variants on Bxb1-mediated recombination activity as displayed as the fold change of the area under the flipping curve (AUC) within different time intervals after induction over a control expressing mCherry instead of TEVp. TEVp variants and designations are derived from a previous study[1], and C151A derivatives are corresponding catalytically inactive mutants. Results from two replicate shake flask cultures (n=1) are shown. (**b**) Contribution of TEVp binding and proteolysis to the DNA recorder output. Displayed are differences in AUC within four hours after induction (ΔAUC_{4h}) relative to a control expressing mCherry instead of TEVp for the two replicate shake flask cultures (n=1) from (a). The binding contributions are the ΔAUC_{4h} of the respective catalytically inactive C151A mutants. The proteolysis-dependent contribution was derived by subtracting the binding contribution from the ΔAUC_{4h} of the active TEVp variants 0 and I, respectively. Source data are provided as a Source Data file.

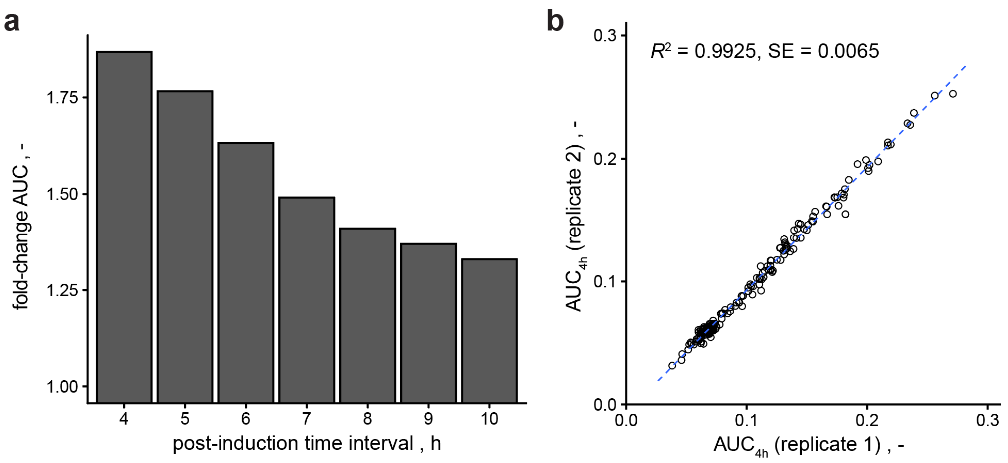

Fig. S4. Evaluation of DNA recorder-derived point metrics for proteolytic activity. (**a**) The fold change of the area under the flipping curve (AUC) of active TEVp 0 over catalytically inactive TEVp 0 C151A is displayed for different time intervals after induction (n=1). (**b**) Analysis of robustness of the AUC_{4h} across experiments. Shown are values for n=181 variants and two independent shake flasks cultivations (circles) with a linear fit (dashed line). R^2: coefficient of determination. SE: standard error. Source data are provided as a Source Data file.

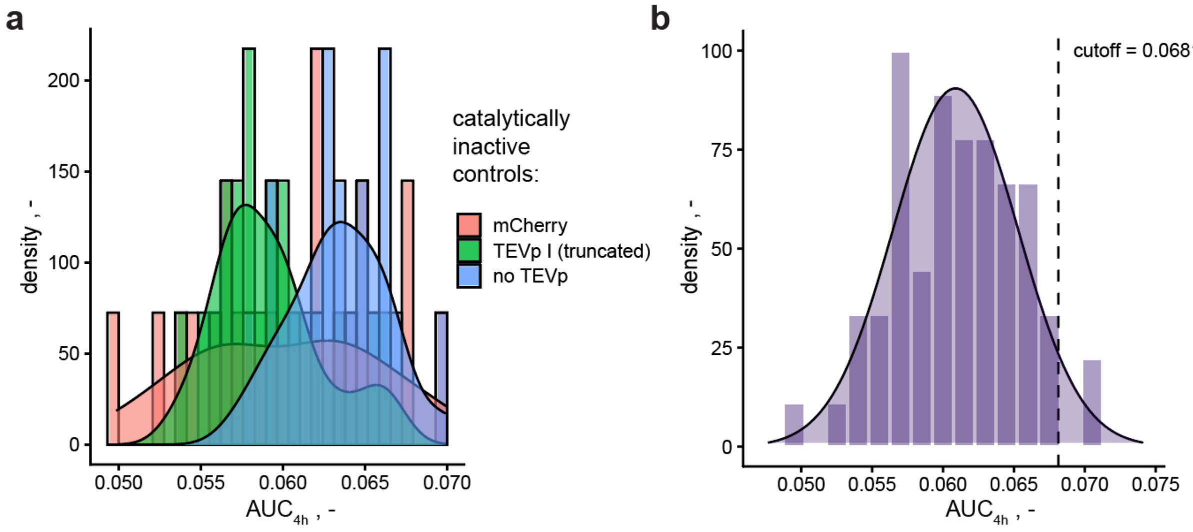

Fig. S5. Correction for binding contribution and experimental noise in DNA recorder data. (**a**) AUC_{4h} density plots (shaded areas) and underlying histograms (bars) for three catalytically inactive controls tested on all 20 P1' variants of TEVs. Controls are clones expressing mCherry instead of TEVp (n=20), a truncated TEVp I (stop codons in position 30 and 31, n=20) and no TEVp (n=20), respectively. A Shapiro-Wilk normality test yielded p-values >0.05 for all three controls ($p_{mCherry}$ = 0.8277, $p_{TEVp\ I,\ trunc.}$ = 0.306, and p_{noTEVp} = 0.9918) indicating that data follow a normal distribution. (**b**) Joint AUC_{4h} histogram (bars) for all controls (n=60) from (a) with a fitted normal distribution (shaded area). The mean of the fitted normal distribution was subtracted from AUC_{4h} of all variants to yield the DNA recorder metric for proteolytic activity. The value under which a control sample falls with a probability of 95% is indicated by a dashed line and was used as cutoff above which variants were considered catalytically active. Source data are provided as a Source Data file.

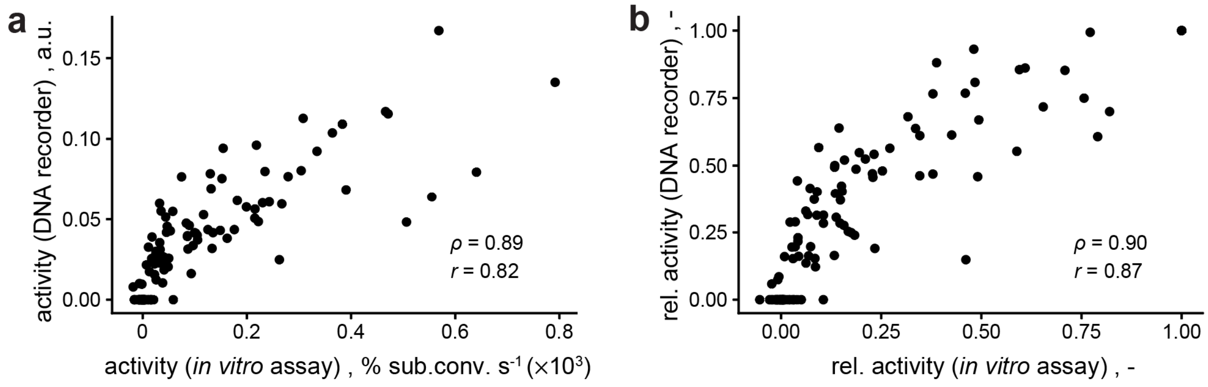

Fig. S6. Correlation between metrics for proteolytic activity derived from the DNA recorder and *in vitro* benchmark assay. Metrics are displayed as absolute values (**a**) or normalized to the activity on the canonical TEVs motif ENLYFQ|S (**b**) for six TEVp variants tested on 20 different substrates (all possible amino acids in P1'). The *in vitro* activity corresponds to the initial reaction rate (within first 15 min) in percent of substrate converted per second (% sub.conv. s^{-1})[1]. Spearman's rank correlation coefficient ρ and Pearson's correlation coefficient r are indicated (n=120). Source data are provided as a Source Data file.

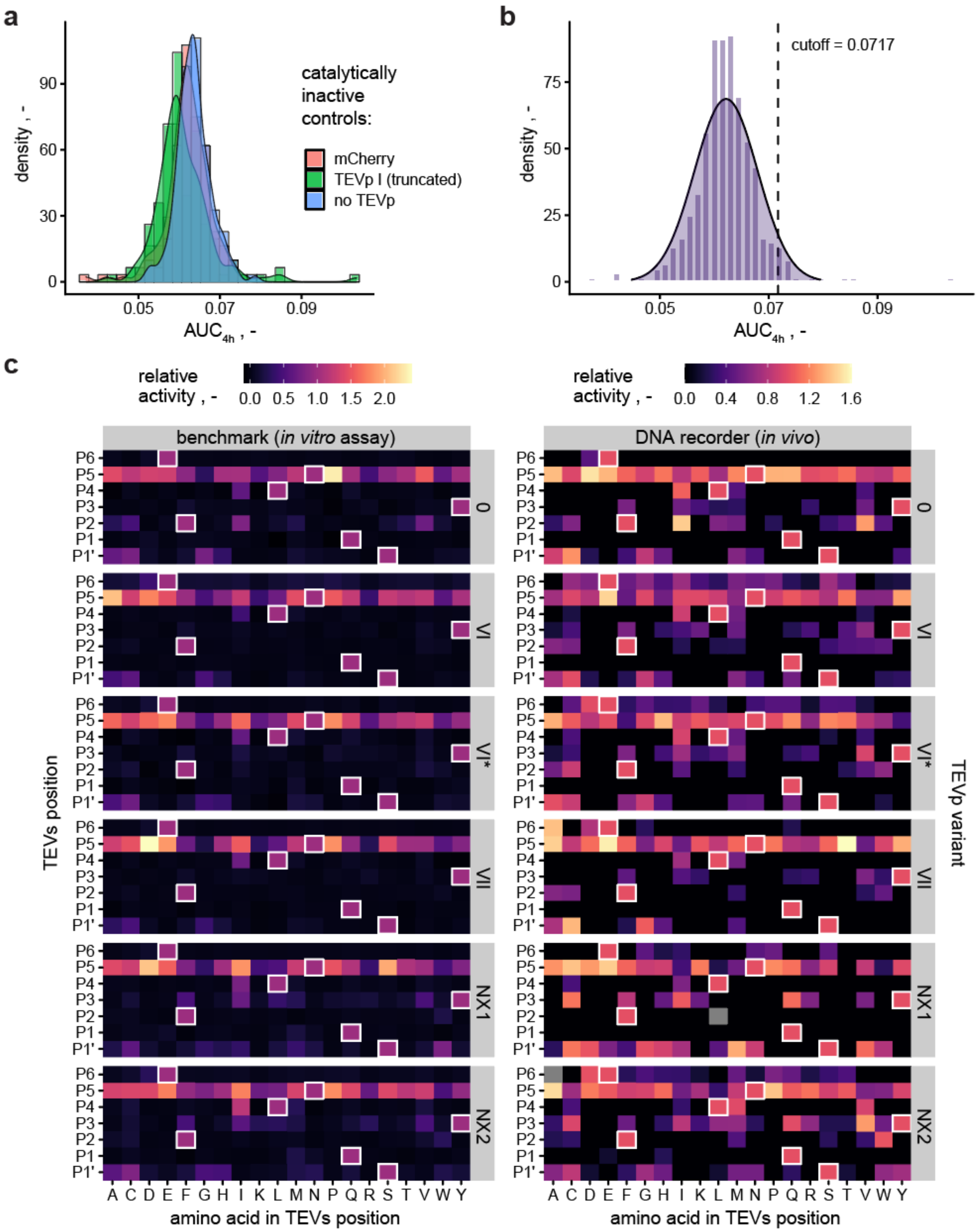

Fig. S7. Correction and benchmarking of DNA recorder data for two-dimensional mutational scan. (**a**) AUC_{4h} density plots (shaded areas) and underlying histograms (bars) for three catalytically inactive controls tested on all 134 single-site mutants of TEVs. Controls are clones expressing mCherry instead of TEVp (n=134), a truncated TEVp I (stop codons in position 30 and 31, n=134) and no TEVp (n=134), respectively. (**b**) Joint AUC_{4h} histogram (bars) for all controls (n=402) from (a) with a fitted normal distribution (shaded area). The mean of the fitted normal distribution was subtracted from AUC_{4h} of all variants from the two-dimensional mutational scan to yield the DNA recorder metric for proteolytic activity. The value under which a control sample falls with a probability of 95% is indicated by a dashed line and was used as cutoff above which variants were considered catalytically active. (**c**) Specificity profiles of six TEVp variants as determined *in vivo* using the DNA recorder (right) or in individual *in vitro* assays (left). Displayed activities (n=1) were normalized to the activity on the canonical TEVs motif for both assays. Amino acids of the canonical TEVs motif are highlighted by white boxes. Gray boxes indicate missing data points. TEVp variants and designations are derived from a previous study[1]. Source data are provided as a Source Data file.

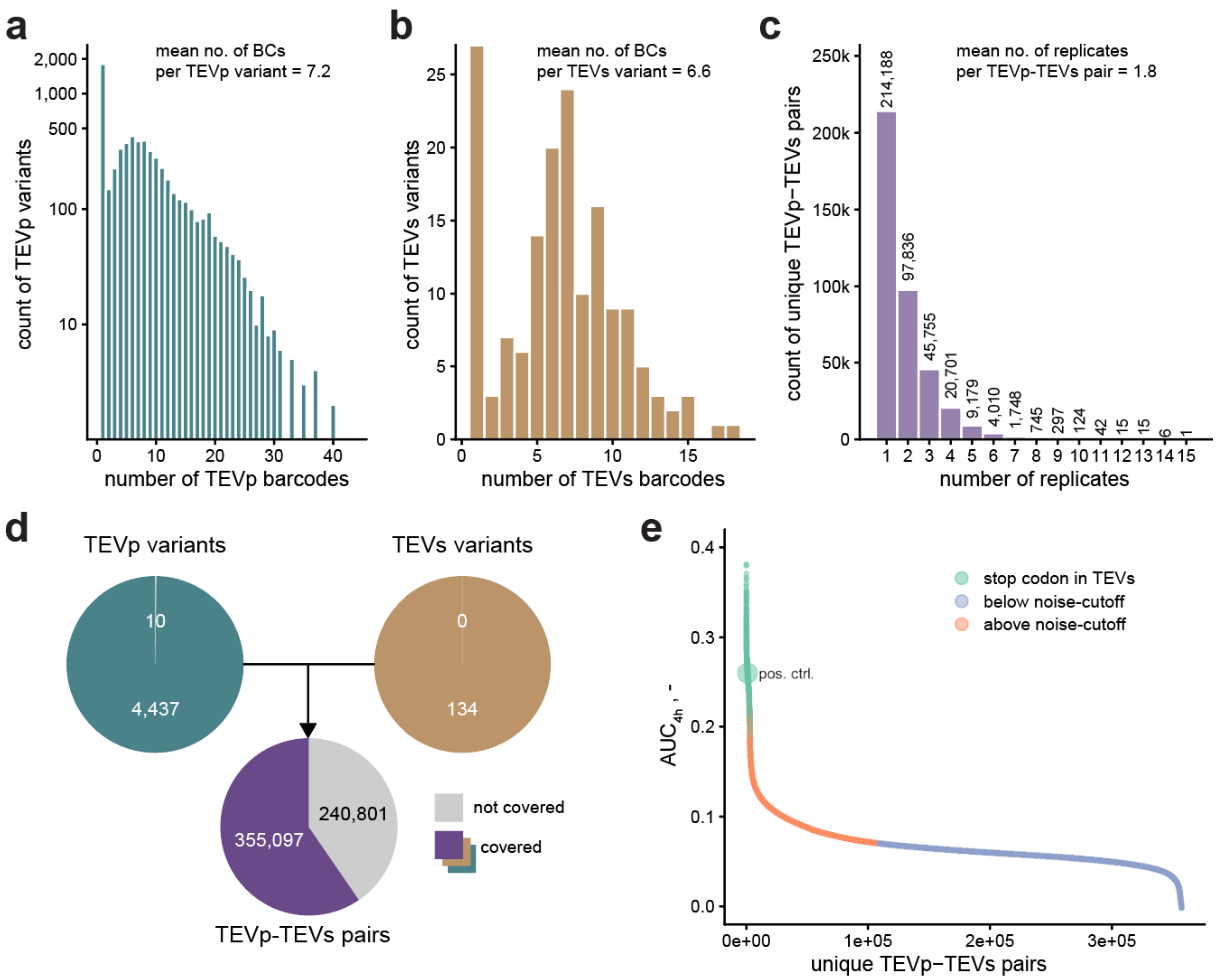

Fig. S8. Variant- and barcode (BC) coverage in the 2D mutational scan library. (**a**) Distribution of the number of BCs per TEVp variant (excluding parent variant TEVp 0). (**b**) Distribution of the number of BCs per TEVs variant (excluding canonical TEVs). (**c**) Distribution of the number of replicates (i.e. unique BC combinations) per unique TEVp-TEVs pair (excluding TEVp 0 and canonical TEVs). (**d**) Coverage within the 2D mutational scan library. (**e**) Distribution of DNA recorder activities (AUC_{4h}) within the library. Individual variants (n=1) are displayed. Source data are provided as a Source Data file.

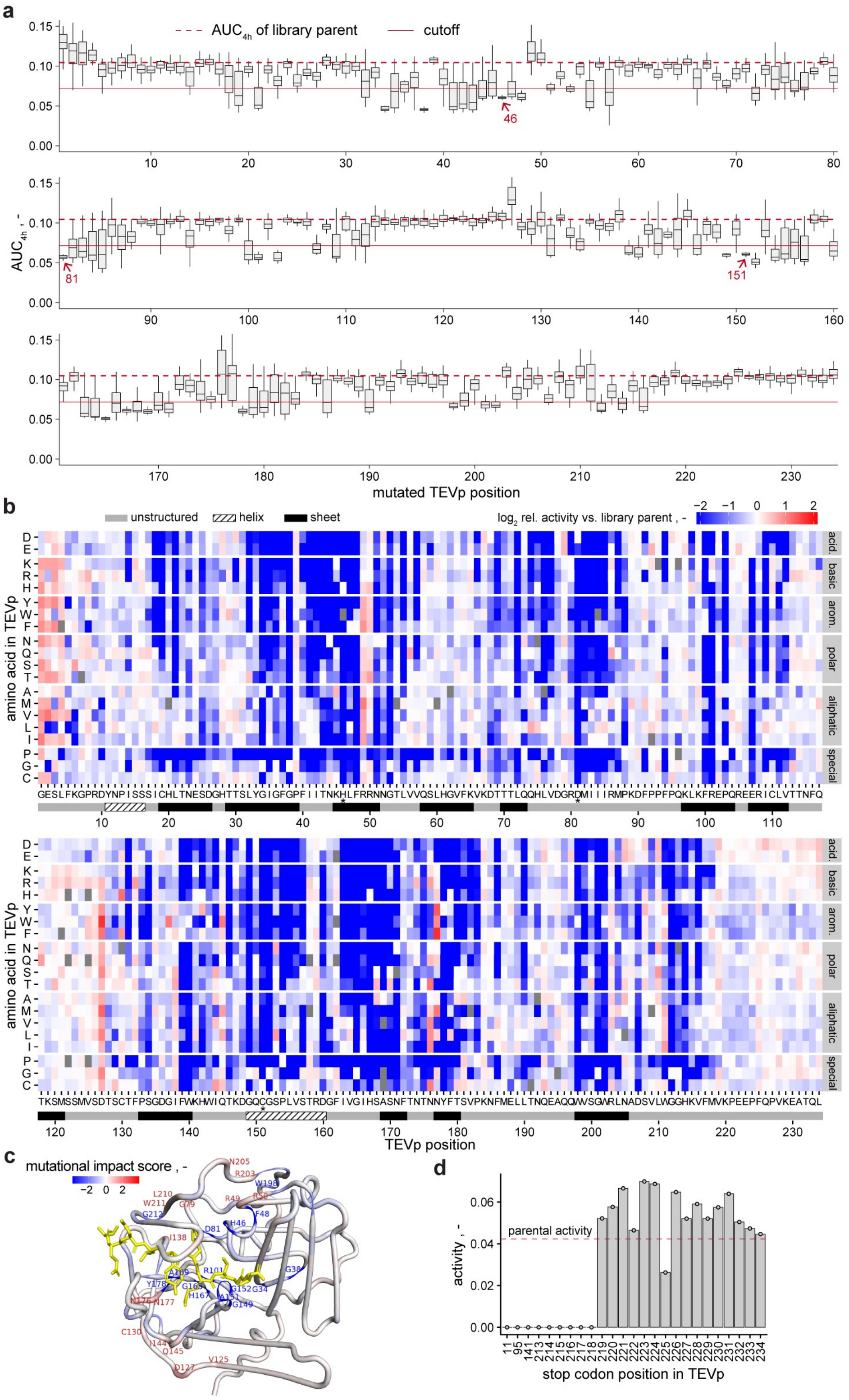

Fig. S9. Mutational scan of TEVp tested on the canonical TEVs motif ENLYFQ|S. (**a**) Positional AUC_{4h} distribution. Boxes outline interquartile range (IQR) with median (horizontal line) and 1.5-fold IQR (whiskers). The AUC_{4h} for the library parent TEVp 0[1] and the applied background cutoff are shown as dashed and solid red lines, respectively. The positions of the catalytic triad are highlighted (red arrows). The number of variants per mutated TEVp position is variable and can be found in the source data file underlying this figure. (**b**) Activities of 4,642 single-site substituents on the canonical TEVs. Activities are displayed as log_2-FC relative to the library parent TEVp 0. Grey boxes indicate missing data. Amino acids are grouped by chemical category and ranked by hydrophobicity within each category. Secondary structure elements are indicated beneath the heatmap and asterisks are catalytic triad positions. (**c**) TEVp structure (PDB 1LVB) in tube representation complexed with substrate peptide (yellow sticks). Tube thickness and color indicate the positional impact of mutations (i.e. activity of the 90th percentile relative to TEVp 0 shown as log_2-FC). The 15 positions with the strongest negative and positive impact score are highlighted in blue and red, respectively. (**d**) Activity of TEVp variants with stop codons at the indicated positions (n=1). Source data are provided as a Source Data file.

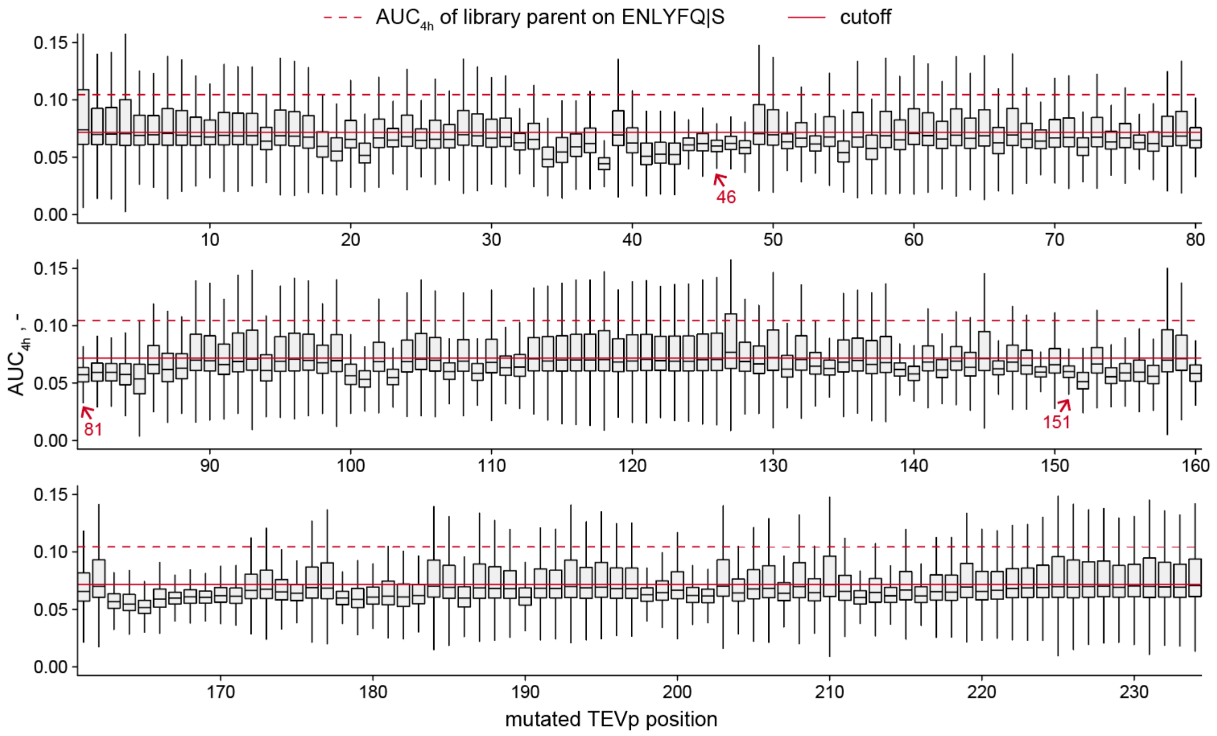

Fig. S10. Mutational scan of TEVp tested on up to 134 single-site variants of TEVs. Positional AUC_{4h} distribution. Boxes outline interquartile range (IQR) with median (horizontal line) and 1.5-fold IQR (whiskers). The AUC_{4h} for the library parent TEVp 0[1] on canonical TEVs and the applied background cutoff are shown as dashed and solid red lines, respectively. The positions of the catalytic triad are highlighted (red arrows). The number of variants per mutated TEVp position is variable and can be found in the source data file underlying this figure. Source data are provided as a Source Data file.

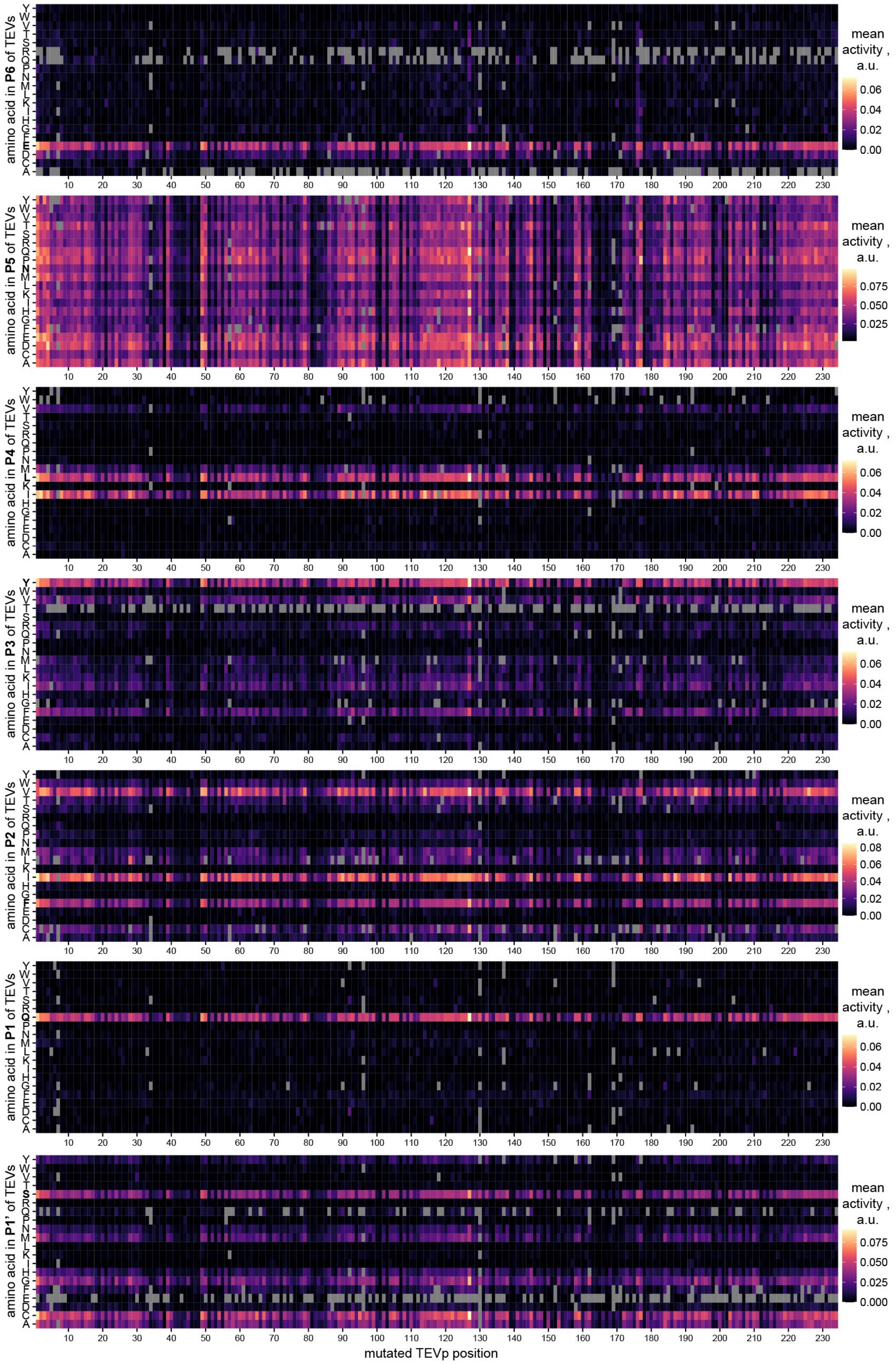

Fig. S11. Mean activity per mutated TEVp position on TEVs variants with mutated positions P6 to P1'. Only cases where at least n=5 TEVp mutants have been tested on the respective TEVs variant are displayed. Gray boxes indicate cases with less than five TEVp mutants tested. Amino acids of the canonical TEVs motif ENLYFQ|S are highlighted in bold. Source data are provided as a Source Data file.

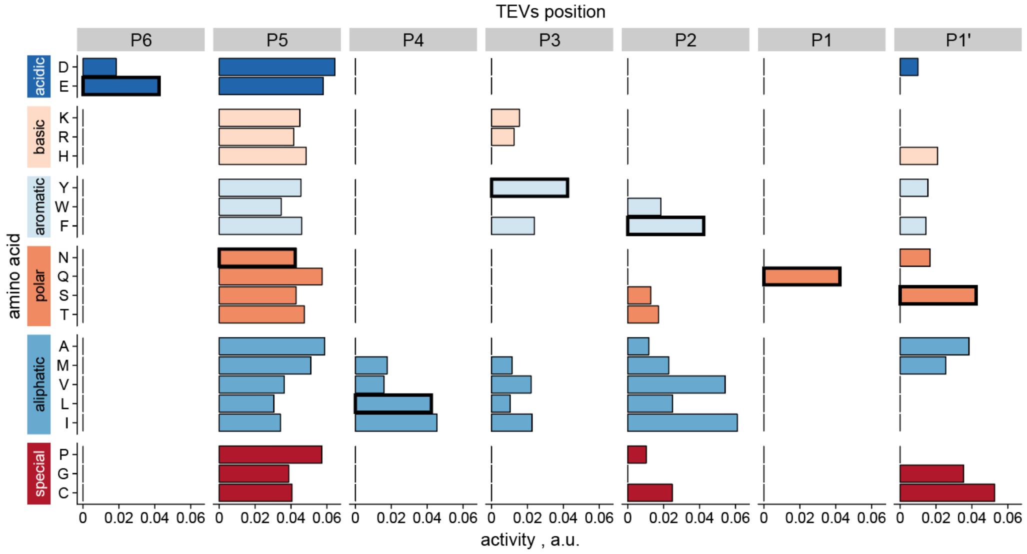

Fig. S12. Activity profile of TEVp 0 on all single-site mutants of the canonical TEVs. Bars show activities on the TEVs variant bearing the indicated amino acid in the respective position (n=1) while for the respective other TEVs positions the canonical amino acids are maintained. Amino acids are grouped by chemical category and ranked by hydrophobicity within each category. The canonical TEVs ENLYFQS is highlighted by thick bar outlines. Source data are provided as a Source Data file.

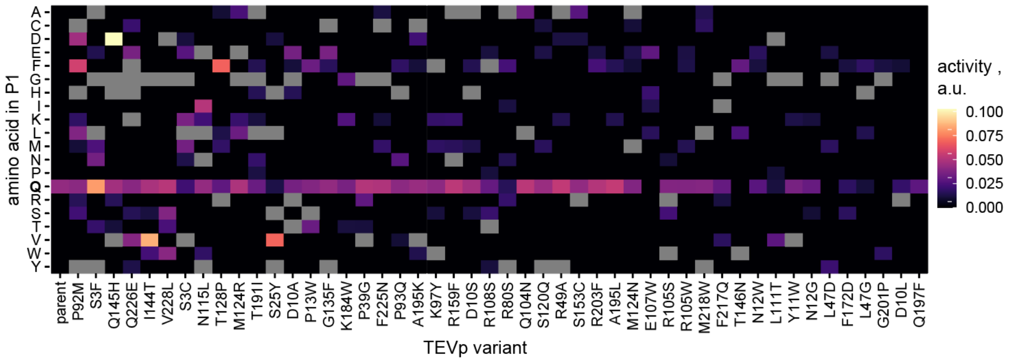

Fig. S13. TEVp variants with high promiscuity at TEVs position P1. Promiscuity was calculated for variants (n=1) that have been tested against at least 15 different P1-variants of TEVs as the Shannon entropy of the AUC_{4h} multiplied by the mean AUC_{4h}. Variants are ranked according to their promiscuity from high (left) to low (right). The parent variant TEVp 0 exhibiting wildtype-like specificity is shown on the left. The canonical amino acid (Q) for P1 in TEVs is highlighted in bold face. Source data are provided as a Source Data file.

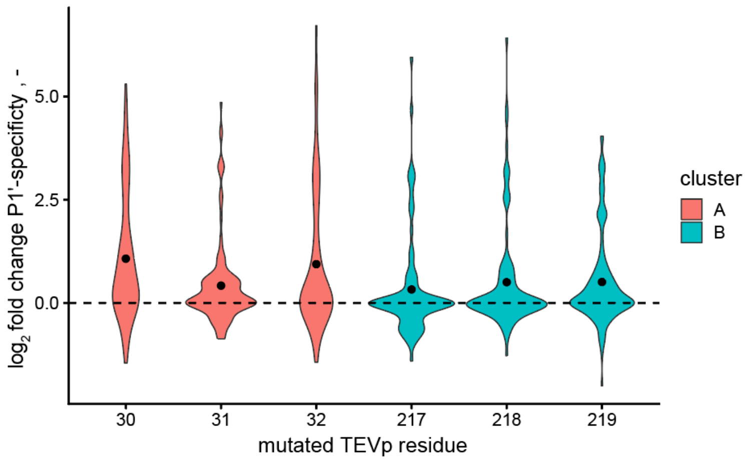

Fig. S14. Impact of single-site mutagenesis of TEVp residues from cluster A and B on P1'-specificity. The fold change in P1'-specificity is calculated as the activity of a variant on a given P1'-mutant of TEVs relative to the canonical TEVs and normalized to the relative activity of the library parent TEVp 0. Black circles are mean fold changes. The number n of variants per mutated TEVp residue is variable and can be found in the source data file underlying this figure. Source data are provided as a Source Data file.

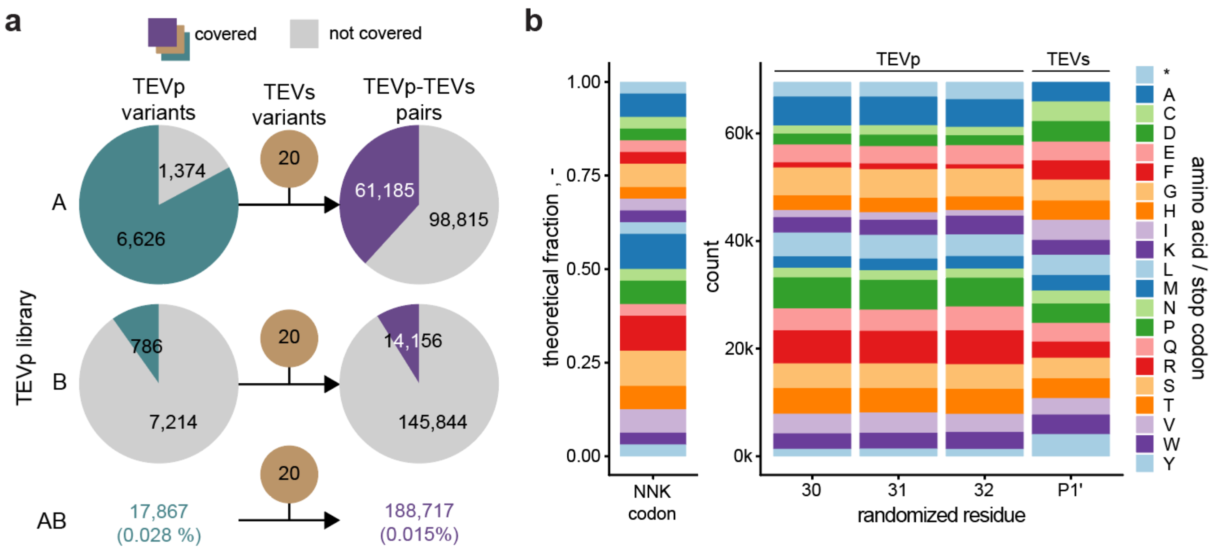

Fig. S15. Analysis of TEVp libraries A, B and AB. (**a**) Coverage of the individual libraries excluding stop codon variants. Percentages indicated the relative coverage of the entire theoretical sequence space for library AB. (**b**) Amino acid distribution in the randomized residues of TEVp and TEVs in library A. Note that the TEVp library was cloned using degenerate codons (NNK) whereas the TEVs library was constructed by cloning variants individually and pooling them in equimolar amounts. Colors in the plot represent amino acids in the same order as shown in the legend. The asterisk denotes stop codons occurring as part of NNK-randomization. For comparison, the theoretical amino acid distribution resulting from NNK is shown (left). Source data are provided as a Source Data file.

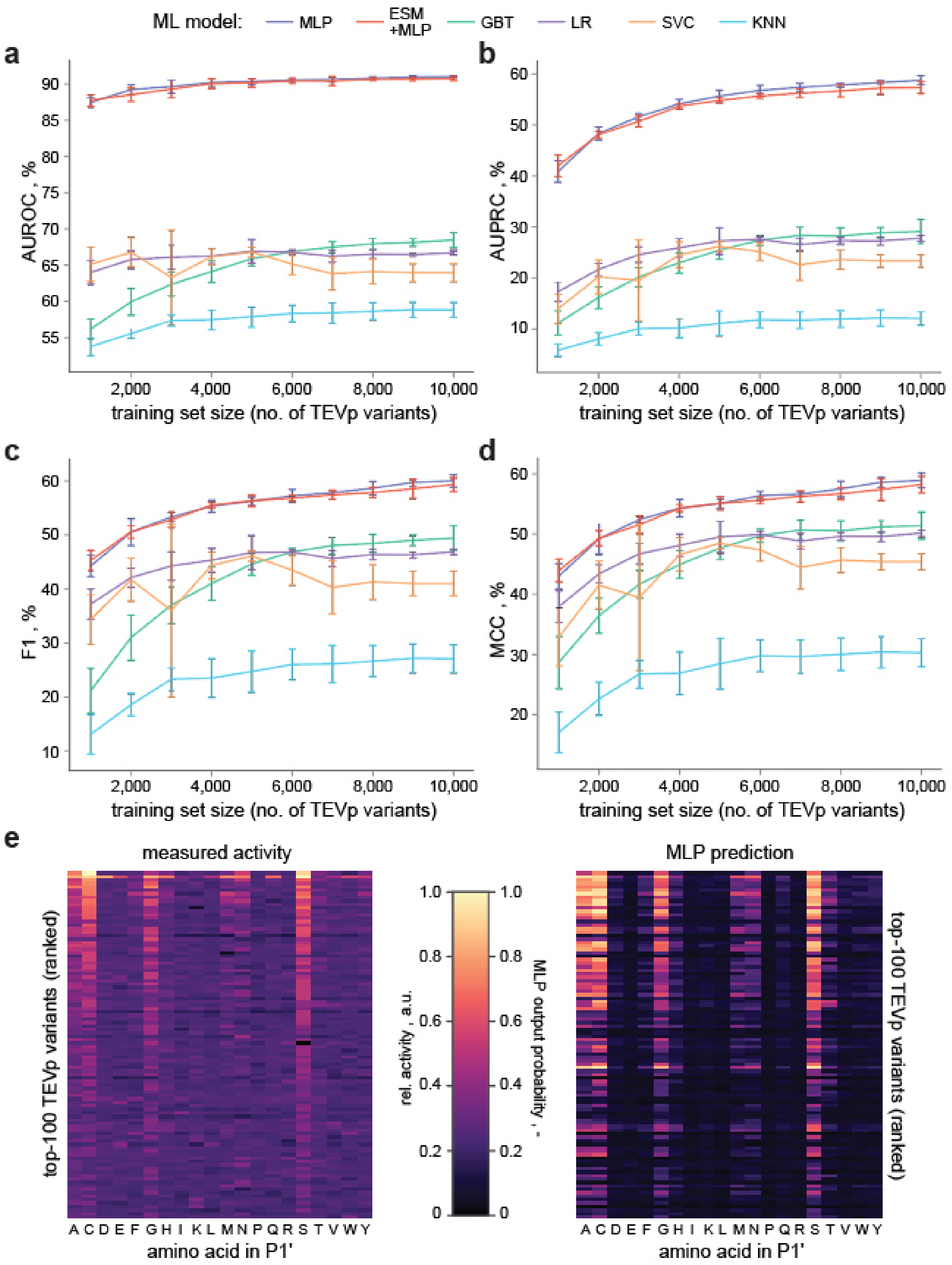

Fig. S16. Dependence of ML model performance on training set size. Four different metrics for prediction accuracy, AUROC (**a**), AUPRC (**b**), F1 (**c**) and MCC (**d**), are displayed. The mean values and standard deviation over n=5 models initialized with different random seeds is shown as error bars. (**e**) Exemplary comparison between measurement and prediction. TEVp variants are ranked according to their maximum activity in the experimental measurement from most (top) to least (bottom) active. Source data are provided as a Source Data file.

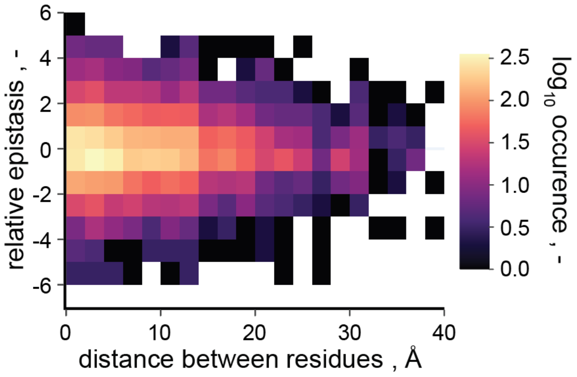

Fig. S17. Epistasis between TEVp residue pairs over their spatial distance (reference structure: 1LVB). Additive epistasis was determined using the additive model described by Olson et al. 2014 (4, Methods) and is displayed in bins of 1 and 2 Å for epistasis and distance, respectively. Source data are provided as a Source Data file.

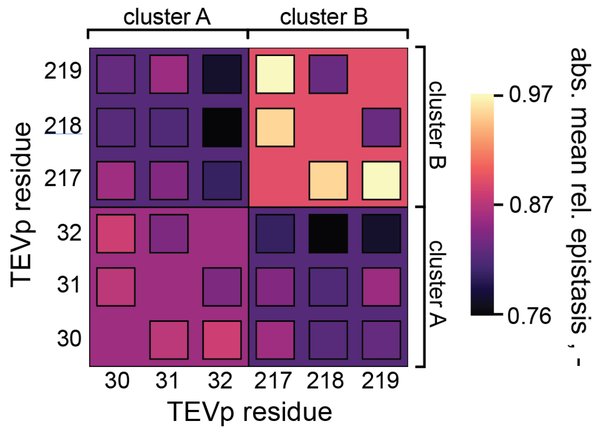

Fig. S18. Additive epistasis in the sequence-activity landscape learned by the MLP model trained on all available data of library AB. Additive epistasis between residues is calculated as in Figure S17 and displayed as the absolute of the mean across residue (small squares) and cluster pairs (large squares), respectively. Source data are provided as a Source Data file.

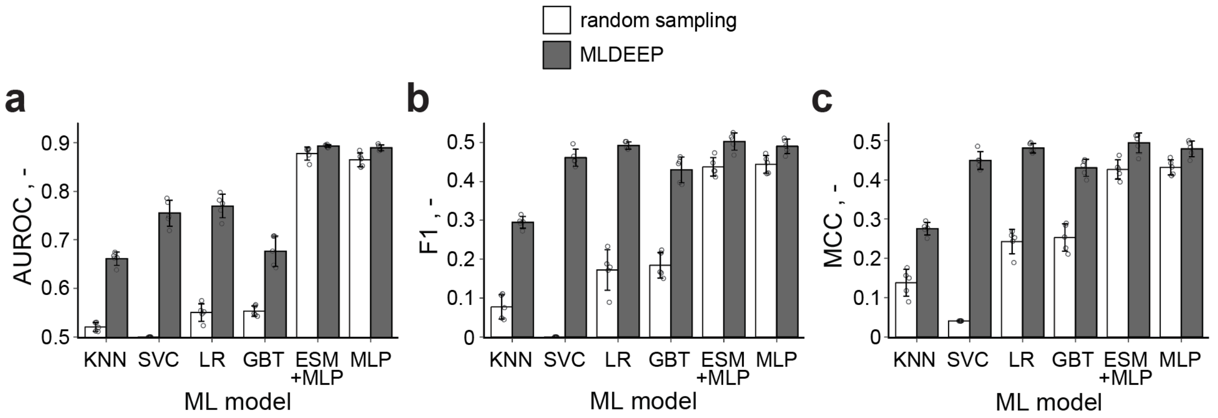

Fig. S19. Comparison of the performance of models resulting from training data obtained by random versus epistasis-inspired (= MLDEEP) sampling. The secondary performance metrics AUROC (**a**), F1 (**b**), and MCC (**c**) are displayed. For random sampling, models were trained with data on 1,000 TEVp variants from library AB. For MLDEEP, models were trained with data on 300 variants each from library A and B as well as 400 variants from library AB. Bars indicate the respective mean performance metric of n=5 replicate models initiated with different random seeds with standard deviation (error bars). Source data are provided as a Source Data file.

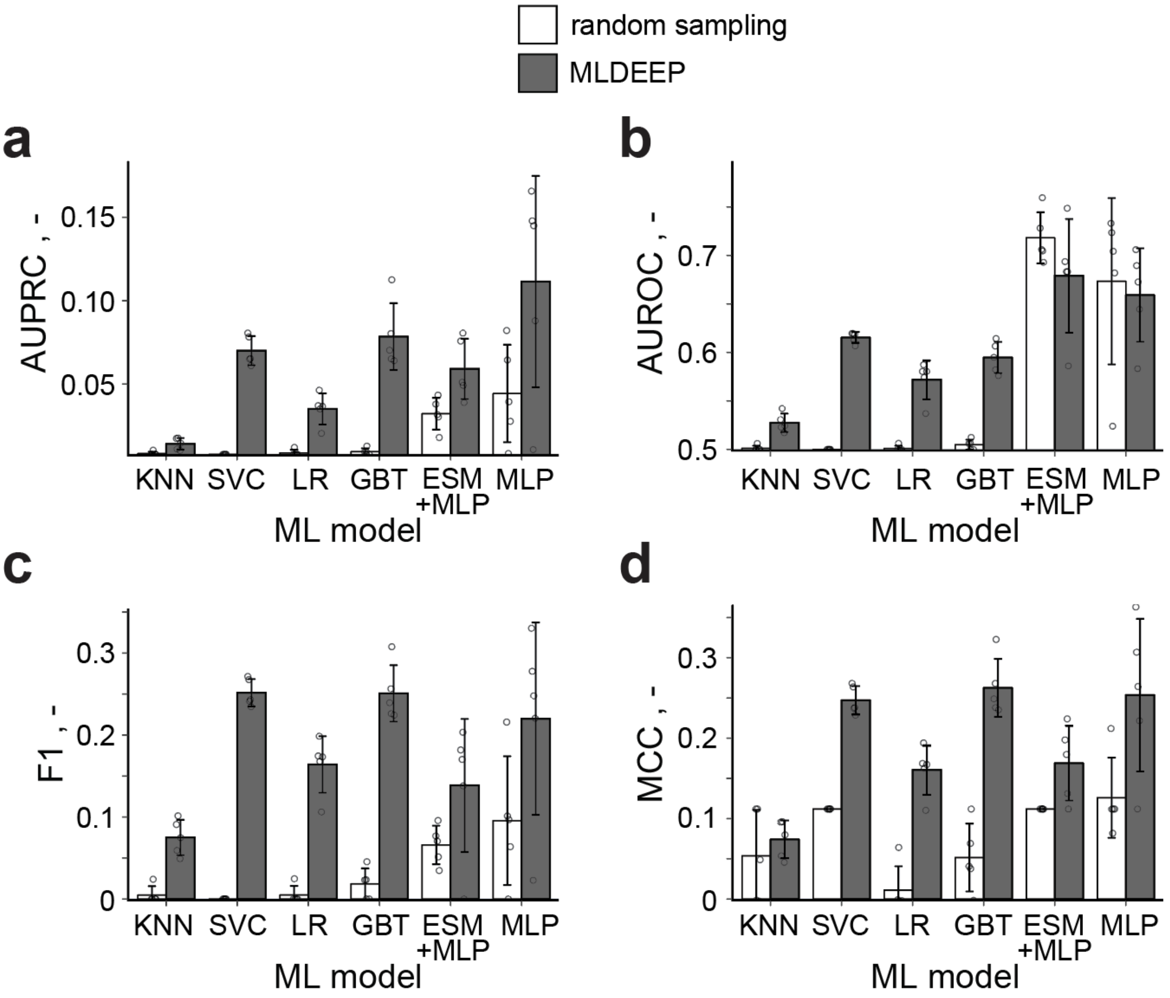

Fig. S20. Comparison of the performance of models resulting from training data obtained by random versus epistasis-inspired (= MLDEEP) sampling for clusters A and C. The performance metrics AUPRC (**a**), AUROC (**b**), F1 (**c**), and MCC (**d**) are displayed. Cluster C corresponds to residues D148, G149 and Q150 of TEVp and data for libraries C and AC were acquired as described before for libraries B and AB. For random sampling, models were trained with data on 1,000 TEVp variants from library AC. Because of fewer completely measured variants in this library, the test and stop set sizes were reduced to 500 and 100, respectively. For MLDEEP, models were trained with data on 300 variants each from library A and C as well as 400 variants from library AC. Bars indicate the respective mean performance metric of n=5 replicate models initiated with different random seeds with standard deviation (error bars). Source data are provided as a Source Data file.

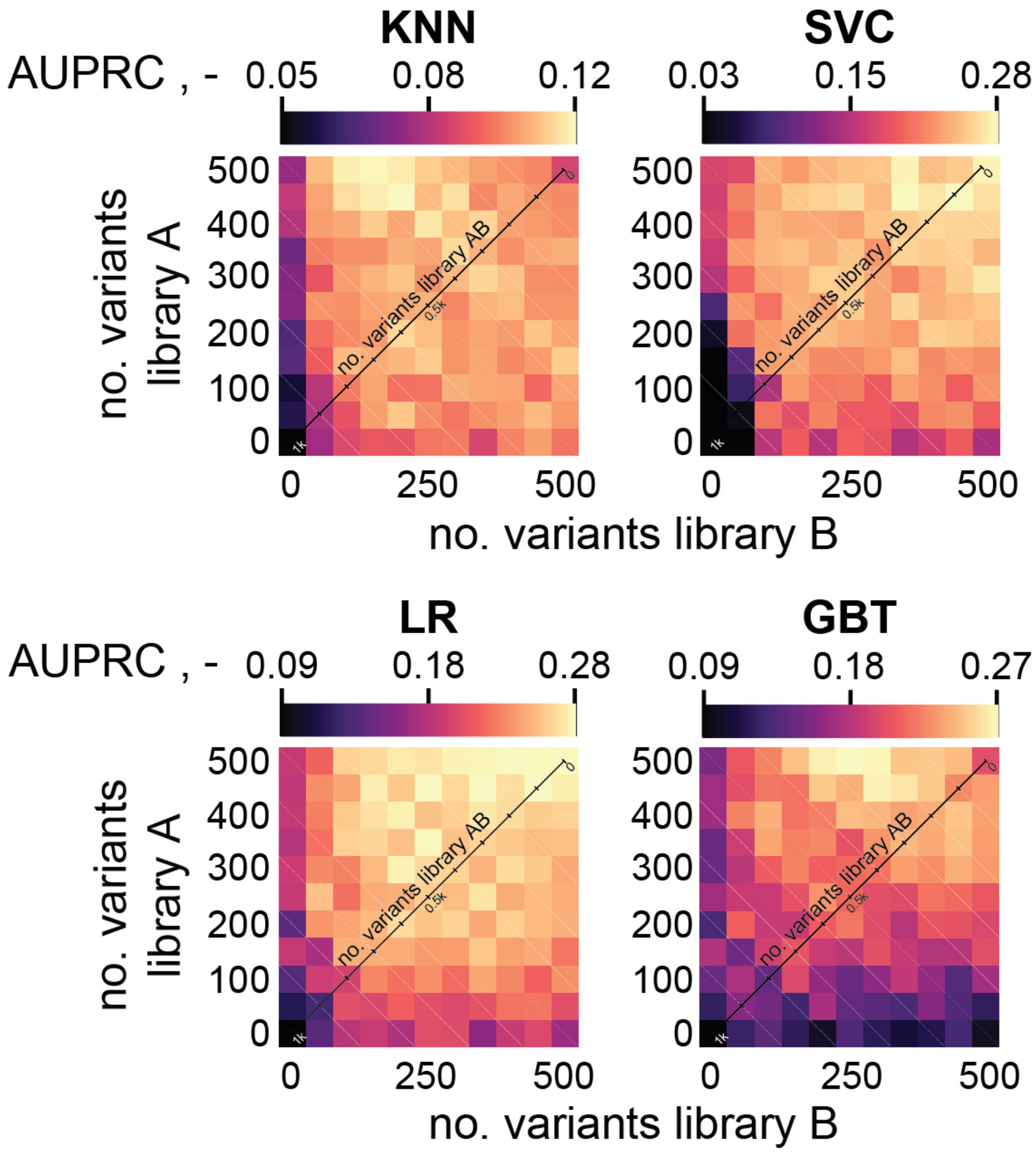

Fig. S21. Analysis of different MLDEEP library compositions. A total experimental budget of 1,000 TEVp variants was distributed in different ratios amongst libraries A, B and AB. The number of samples from library AB is indicated on the diagonal axis, i.e. the lower left and upper right squares correspond to 1,000 or no samples from library AB, respectively. Performance of KNN, SVC, LR and GBT models is displayed as the mean AUPRC of n=5 replicate models initiated with different random seeds. Source data are provided as a Source Data file.

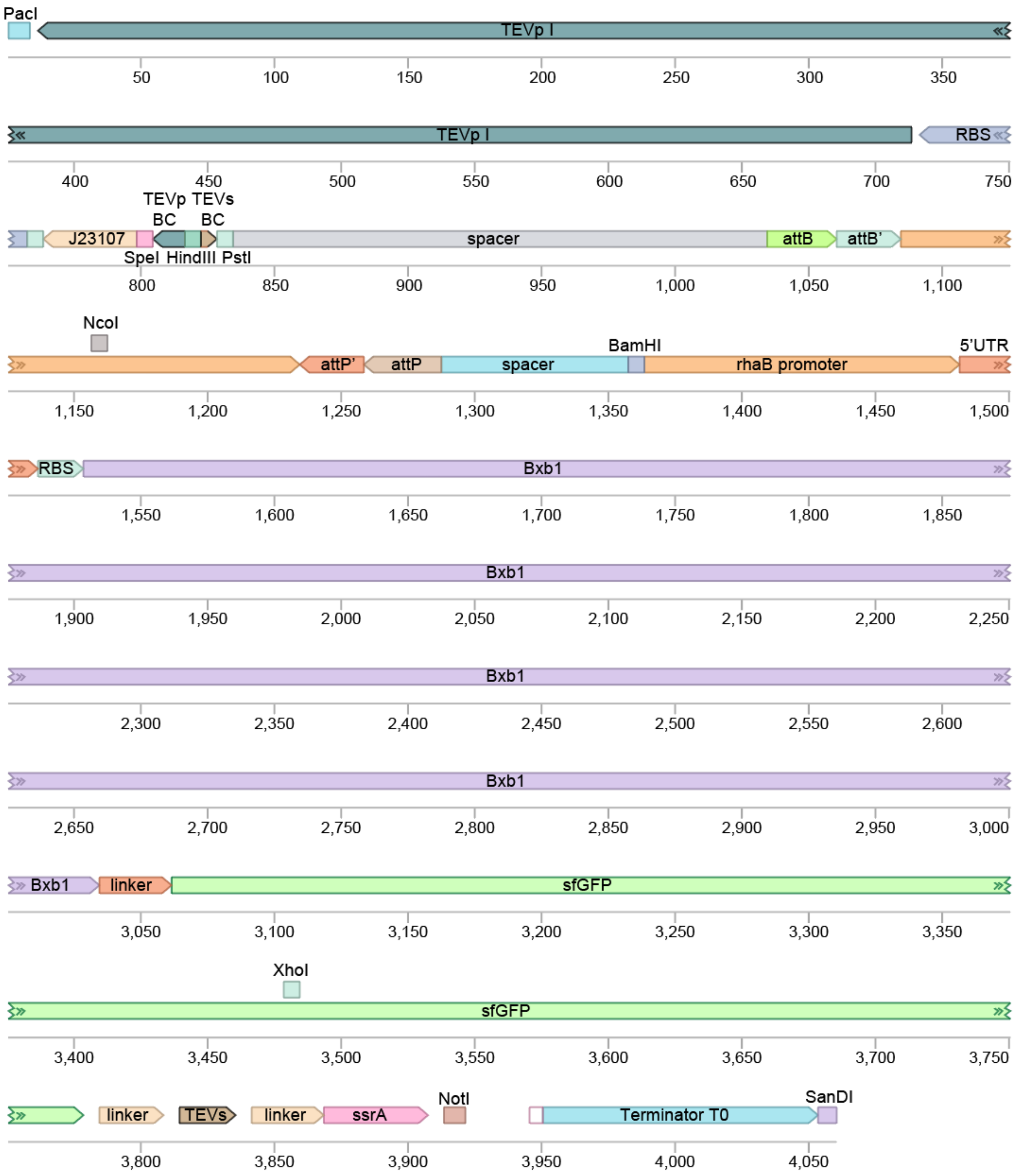

Fig. S22. Plasmid map of pProtRec. The region from *Spe*I to *Nco*I is isolated as target fragment for paired-end NGS, and contains barcodes specific for the respective TEVp and TEVs variants as well as a part of the Bxb1 substrate (here denoted as discriminator) flanked by *attB/attP* recombination sites. The pSEVA291 sequence from SanDI to PacI used as a plasmid backbone is omitted. The nucleotide sequence underlying this map is provided in **Supplementary note 1**.

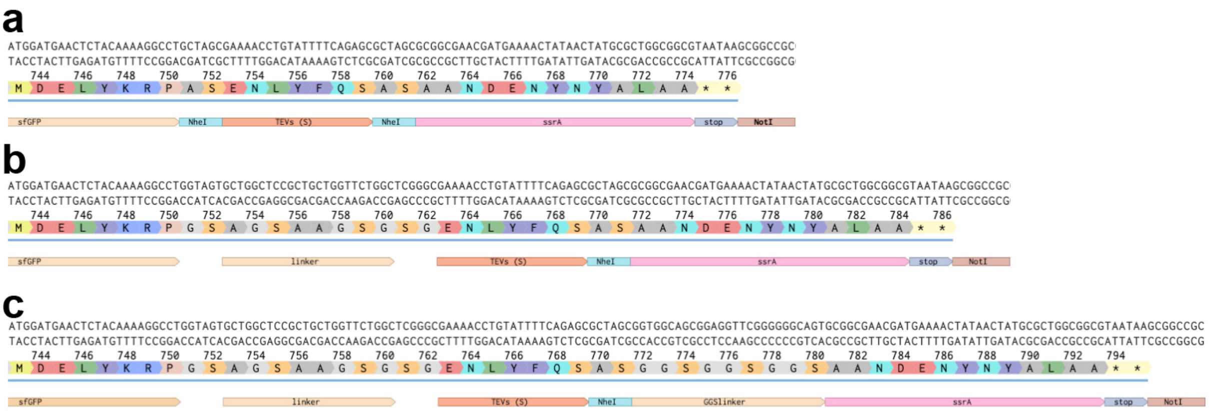

Fig. S23. Maps of different linkers flanking *tevs*. TEVs is placed between the Bxb1-sfGFP fusion and the SsrA-tag. Different versions with no (**a**)**,** one (**b**) and two (**c**) linkers are displayed (region from 3'-end of *sfGFP* to *Not*I only). Plasmid pProtRec corresponds to the best-performing version with two linkers (**c**). Sequences of the displayed constructs are provided in **Supplementary notes 7-9**.

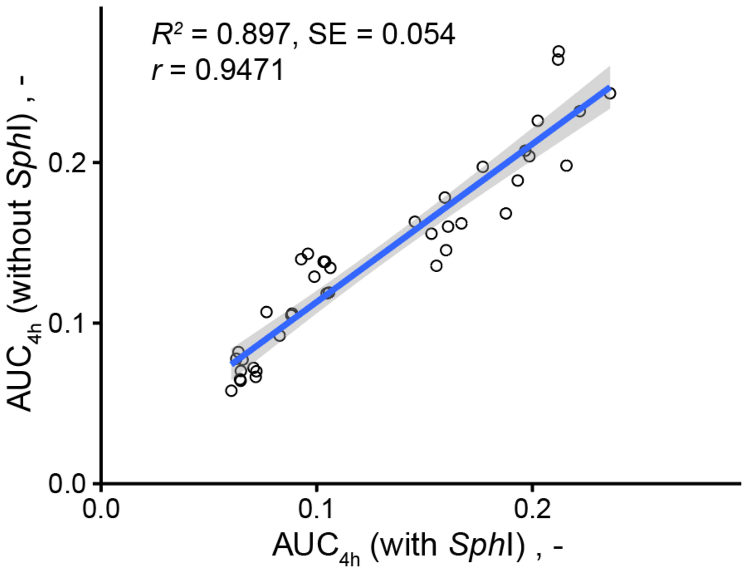

Fig. S24. Comparison DNA recorder output (AUC$_{4h}$) with and without an *Sph*I site downstream of *tevs*. To facilitate cloning of the TEVs single-site variants library, the recorder plasmid was adapted introducing an *Sph*I restriction site between *tevs* and *ssrA*. This modification does not significantly affect the DNA recorders output as shown for different internal controls (n=40) tested with the canonical TEVs. Blue line: linear correlation. R^2, coefficient of determination; SE, standard error; r, Pearson's correlation coefficient. Source data are provided as a Source Data file.

Supplementary tables

Tab. S1. TEVp residues interacting with the canonical substrate ENLYFQ|S and their mutational impact on activity. Bold residues form hydrogen-bonds, others form hydrophobic interactions with the respective substrate positions as previously reported[5]. Residues are colored according to their impact on activity upon mutation (see Tab. S2 and S3) with red ones being amongst the 15 positions with strongest positive and blue ones being amongst the 30 positions with the strongest negative effect.

**TEVs position:**	**P6**	**P5**	**P4**	**P3**	**P2**	**P1**	**P1'**
**interacting TEVp residues**	**N171**<br>**N176**<br>**Y178**	-	A169<br>N171<br>Y178<br>H214	**D148**<br>**N174**<br>V216<br>K220	H46<br>V209<br>W211<br>V216<br>M218	**T146**<br>**D148**<br>**H167**<br>S170	H46

Tab. S2. Top 25 TEVp residues with strongest negative mutational impact on the activity towards canonical TEVs. Shown are the 25 top positions with the lowest mutational impact score ranked from low to high. The mutational impact score is defined as the 90th percentile activity of mutants relative to the library parent TEVp 0 (i.e. values below 1.0 indicate a tendency to decrease activity compared to TEVp 0 upon mutation). Positions of the catalytic triad are highlighted in red. Top and mean activities are shown relative to TEVp 0. aa: amino acid.

TEVp pos.	parent aa	mutants tested	mutants above cutoff	top activity [a.u.]	top aa	mut. impact score	mean activity [a.u.]	comments
38	G	19	0	0	-	0	0	-
46	H	18	0	0	-	0	0	hydrophobic interaction with S in P1’
48	F	19	1	0.802	W	0	0.042	deeply embedded, part of helical structure hosting H46
81	D	19	1	0.315	E	0	0.017	-
101	R	19	1	1.203	K	0	0.063	-
149	G	19	0	0	-	0	0	-
152	G	19	0	0	-	0	0	-
165	G	19	0	0	-	0	0	-
167	H	19	0	0	-	0	0	H-bond to Q in P1
169	A	16	0	0	-	0	0	hydrophobic interaction with L at P4
178	Y	19	1	0.662	F	0	0.035	hydrophobic interaction with L in P4, H-bond with E in P6
151	C	19	2	0.334	S	0.065	0.035	-
34	G	19	2	0.917	A	0.073	0.068	-
198	W	19	3	0.445	F	0.264	0.056	-
212	G	19	3	0.457	A	0.275	0.055	-
53	G	19	8	0.362	T	0.303	0.118	-
171	N	18	3	0.556	D	0.304	0.068	hydrophobic interaction with L in P4, H-bond with E in P6
202	W	19	4	0.53	Y	0.322	0.081	-
140	W	19	4	0.615	Y	0.371	0.09	-
201	G	19	4	0.424	A	0.373	0.08	-
107	E	19	7	1.014	D	0.379	0.155	-
51	N	19	9	0.407	E	0.399	0.162	-
175	T	19	13	0.537	S	0.452	0.244	between N174 and N176, which interact with TEVs
82	M	19	6	0.797	L	0.465	0.153	-
214	H	19	8	0.535	F	0.476	0.16	hydrophobic interaction with L in P4

Tab S3. Top 25 TEVp residues with strongest positive mutational impact on the activity towards canonical TEVs. Shown are the 25 positions with the highest mutational impact score ranked from high to low. The mutational impact score is defined as the 90th percentile activity of mutants relative to TEVp 0 (i.e. values greater than 1.0 indicate a tendency to increase activity upon mutation). Top and mean activities are shown relative to TEVp 0. aa: amino acid.

**TEVp pos.**	**parent aa**	**mutants tested**	**mutants above cutoff**	**top activity [a.u.]**	**top aa**	**mut. impact score**	**mean activity [a.u.]**	**comments**
127	D	19	19	3.291	W	2.281	1.731	surface exposed in loop distant from substrate tunnel
177	N	19	14	3.345	F	2.256	1.069	between N176 and Y178, which interact with TEVs
176	N	19	18	2.211	T	1.996	1.103	H-bond with E in P6
1	G	19	19	2.077	I	1.909	1.565	5'-end of CDS
3	S	19	19	1.954	Y	1.767	1.228	5'-end of CDS
49	R	19	19	2.117	M	1.749	1.33	surface exposed
2	E	19	17	2.183	V	1.713	1.224	5'-end of CDS
145	Q	19	19	2.122	W	1.627	1.183	surface exposed
4	L	18	17	1.581	K	1.534	1.204	5'-end of CDS
210	L	19	19	1.798	S	1.485	1.086	surface exposed
144	I	19	12	1.772	C	1.438	0.612	surface exposed
138	I	19	19	1.631	T	1.434	1.126	surface exposed, close to S135, S135G increases solubility
130	C	18	18	2.273	H	1.42	1.086	surface exposed, close to S135, S135G increases solubility
211	W	19	15	1.758	I	1.42	0.736	hydrophobic interaction with F in P2
205	N	18	18	1.47	E	1.41	0.901	surface exposed
50	R	19	18	1.694	T	1.367	1.089	close to L56, L56V increases solubility
125	V	16	16	1.375	F	1.342	1.064	surface exposed in loop distant from substrate tunnel
203	R	19	18	1.383	V	1.337	1.062	surface exposed
79	G	19	19	1.501	E	1.331	1.043	surface exposed, close to I77, I77V increases solubility
219	V	19	19	1.418	D	1.324	1.058	S219V and S219D abolish auto-inactivation[6]
234	L	19	19	1.439	D	1.309	1.054	C-terminus
132	F	19	18	1.763	R	1.307	1.028	surface exposed, close to S135, S135G increases solubility
208	S	19	19	1.482	Y	1.304	0.961	surface exposed
8	P	19	18	1.536	R	1.283	0.98	5'-end of CDS
193	Q	19	19	1.463	C	1.261	1.059	surface exposed

Table S4. Top 10 variants yielding highest activities with the respective P1-substitution mutants of TEVs.

P1	rank									
**aa**	**1st**	**2nd**	**3rd**	**4th**	**5th**	**6th**	**7th**	**8th**	**9th**	**10th**
**A**	V63C	T22Y	L32I	K89G	I84V	S208A	G1D	R108Q	G149D	S17P
**C**	F116C	K184A	V219H	S129V	S157T	K89F	L4D	G62M	V156A	F225D
**D**	V77T	Q145H	E188N	K97A	P92E	P92M	N12T	G53Y	S208V	Q104S
**E**	P93F	E102L	F64K	S3R	G79N	E2D	P39A	L189M	L32N	L234S
**F**	M82P	F162R	T128P	M87C	P92M	R9N	K6M	Q196Y	L234F	Q197M
**G**	N52E	P103V	K184E	S153N	L76E	H214G	V216W	T70L	T180I	K184W
**H**	A206E	I83K	S200R	I109C	G7H	G1F	N177L	H75K	K97C	L72P
**I**	K6A	N115L	V63L	E102N	I166E	I109A	K184H	K97W	L204D	D26G
**K**	N174P	R203C	R50V	F172V	L210M	G201Y	D26G	F64M	I85M	G152D
**L**	E222L	T71I	V112Y	N51L	K89C	F64Y	F139Y	P92M	P13I	S200L
**M**	K99S	V219E	T232F	G7Q	T69P	F91M	S3V	V199P	R105L	P93G
**N**	S126H	S129V	E102G	S15F	W198R	F225P	Y11T	K141E	Q117M	N44L
**P**	I163F	L204D	N52I	T114P	R86Y	F5A	K141N	R203W	F132R	S168A
**R**	S123E	D10R	K215Y	N185K	N171H	E188P	F91M	F116T	G137V	T180E
**S**	M124H	D136V	V66Q	C151T	T113W	P221F	Q104I	F186C	P92G	S153I
**T**	E24H	L4T	D81N	K97P	T70E	S200K	T29G	M218F	K229S	A206V
**V**	I144T	S25Y	Q117I	N185L	V219G	M82L	Q226E	Q104R	E188I	P39Y
**W**	S126Q	S200G	V209L	D78Q	G201T	Q145P	V228L	P183V	R159S	R9N
**Y**	L55R	I109M	K89R	K147G	F100L	F40V	L210N	P39F	S3M	D78H

Table S5. **Plasmids used in this study.** Sequences of plasmids from this study can be found in Supplementary notes.

**Plasmid**	**Description**	**Source/Reference**
pSEVA291	Basis of DNA recorder plasmid series from this study. Contains a kanamycin resistance cassette, a pBR322 origin of replication and a multiple cloning site	[7]
pASPIre3	Derivative of pSEVA291 containing the Bxb1 substrate and the Bxb1-sfGFP-SsrA fusion under control of P_{rha}	[2], Addgene #154844
pASPIre4	Derivative of pASPIre3 with modified Bxb1 substrate module harboring only one *Nco*I site as used in the DNA recorders from this study.	[8], Addgene #196656
pProtRec	Derivative of pASPIre4. Plasmid embodying the optimized DNA recorder for proteolytic activity used in this study. Contains CDS of TEVp I controlled by constitutive promoter J23107 and a Bxb1-sfGFP-TEVs-$SsrA^{NYNY}$ fusion controlled by a weak RBS. TEVs is flanked by flexible amino acid linkers.	This study
pEXPrfp	Used for the expression of mCherry-TEVp fusion proteins to generate cell free extracts for *in vitro* assays, containing a kanamycin resistance cassette and a p15A origin of replication.	[1]
pFRET	Encoding *mtfp1-tevs-syfp2*. Used for the expression of CFP-YFP fusion proteins with TEVs in-between to generate FRET substrates for *in vitro* assays, containing a kanamycin resistance cassette and a p15A origin of replication.	[1]

Table S6. Primers used to clone P1’-variants of TEVs. Primers used to mutate the P1’-codon (uppercase, reverse complement) are designated TEVs_ENLYFQX. Primers B001-B020 were used to introduce 6-mer barcodes (uppercase) specific for each P1’ variant. TEVs primers are listed in the same order as the corresponding barcoding primers (i.e. B001 belongs to TEVs_ENLYFQS, B002 belongs to TEVs_ENLYFQA, etc.).

**Name**	**Sequence (5’ → 3’)**
TEVs_ENLYFQS	ccgaacctccgctgccaccgctagcGCTctgaaaatacaggttttcgcccg
TEVs_ENLYFQA	ccgaacctccgctgccaccgctagcCGCctgaaaatacaggttttcgcccg
TEVs_ENLYFQC	ccgaacctccgctgccaccgctagcGCActgaaaatacaggttttcgcccg
TEVs_ENLYFQD	ccgaacctccgctgccaccgctagcATCctgaaaatacaggttttcgcccg
TEVs_ENLYFQE	ccgaacctccgctgccaccgctagcTTCctgaaaatacaggttttcgcccg
TEVs_ENLYFQF	ccgaacctccgctgccaccgctagcAAActgaaaatacaggttttcgcccg
TEVs_ENLYFQG	ccgaacctccgctgccaccgctagcGCCctgaaaatacaggttttcgcccg
TEVs_ENLYFQH	ccgaacctccgctgccaccgctagcATGctgaaaatacaggttttcgcccg
TEVs_ENLYFQI	ccgaacctccgctgccaccgctagcAATctgaaaatacaggttttcgcccg
TEVs_ENLYFQK	ccgaacctccgctgccaccgctagcTTTctgaaaatacaggttttcgcccg
TEVs_ENLYFQL	ccgaacctccgctgccaccgctagcCAGctgaaaatacaggttttcgcccg
TEVs_ENLYFQM	ccgaacctccgctgccaccgctagcCATctgaaaatacaggttttcgcccg
TEVs_ENLYFQN	ccgaacctccgctgccaccgctagcGTTctgaaaatacaggttttcgcccg
TEVs_ENLYFQP	ccgaacctccgctgccaccgctagcCGGctgaaaatacaggttttcgcccg
TEVs_ENLYFQQ	ccgaacctccgctgccaccgctagcCTGctgaaaatacaggttttcgcccg
TEVs_ENLYFQR	ccgaacctccgctgccaccgctagcGCGctgaaaatacaggttttcgcccg
TEVs_ENLYFQT	ccgaacctccgctgccaccgctagcGGTctgaaaatacaggttttcgcccg
TEVs_ENLYFQW	ccgaacctccgctgccaccgctagcCCActgaaaatacaggttttcgcccg
TEVs_ENLYFQV	ccgaacctccgctgccaccgctagcCACctgaaaatacaggttttcgcccg
TEVs_ENLYFQY	ccgaacctccgctgccaccgctagcATActgaaaatacaggttttcgcccg
B001	cgttcccctaatcctttcactgcagACATCGaagcttcggtaggctgctac
B002	cgttcccctaatcctttcactgcagTGGTCAaagcttcggtaggctgctac
B003	cgttcccctaatcctttcactgcagCACTGTaagcttcggtaggctgctac
B004	cgttcccctaatcctttcactgcagATTGGCaagcttcggtaggctgctac
B005	cgttcccctaatcctttcactgcagGATCTGaagcttcggtaggctgctac
B006	cgttcccctaatcctttcactgcagTACAAGaagcttcggtaggctgctac
B007	cgttcccctaatcctttcactgcagCGTGATaagcttcggtaggctgctac
B008	cgttcccctaatcctttcactgcagGCCTAAaagcttcggtaggctgctac
B009	cgttcccctaatcctttcactgcagTCAAGTaagcttcggtaggctgctac
B010	cgttcccctaatcctttcactgcagCTGATCaagcttcggtaggctgctac
B011	cgttcccctaatcctttcactgcagAAGCTAaagcttcggtaggctgctac
B012	cgttcccctaatcctttcactgcagGTAGCCaagcttcggtaggctgctac
B013	cgttcccctaatcctttcactgcagTTGACTaagcttcggtaggctgctac
B014	cgttcccctaatcctttcactgcagGGAACTaagcttcggtaggctgctac
B015	cgttcccctaatcctttcactgcagTGACATaagcttcggtaggctgctac
B016	cgttcccctaatcctttcactgcagGGACGGaagcttcggtaggctgctac
B017	cgttcccctaatcctttcactgcagCTCTACaagcttcggtaggctgctac
B018	cgttcccctaatcctttcactgcagGCGGACaagcttcggtaggctgctac
B019	cgttcccctaatcctttcactgcagTTTCACaagcttcggtaggctgctac
B020	cgttcccctaatcctttcactgcagGGCCACaagcttcggtaggctgctac

Table S7. Primers used to generate the TEVs single-site variant library. Randomized barcode region and codons are highlighted in uppercase. The annealing region of the oligo pool is highlighted in italics. [NNN] = equimolar mix of codons encoding all 20 amino acids. [NNN*] = equimolar mix of codons encoding all 20 amino acids and one stop codon.

Name	Sequence (5' → 3')
TEVs_BC_10N	ataaagcttNNNNNNNNNNctgcagtgaaaggattagg
Oligo pool	accgcatgcgctagc[NNN][NNN][NNN][NNN][NNN][NNN][NNN*]gcccgagccagaaccagcagcggagcca gcactaccagg*ccttttgtagagttcatccatgc*

Table S8. Primers used to amplify and barcode (N_{15}-mer) the synthetic TEVp single-site variant library.

Name	Sequence (5' → 3')
TEVp_BC_15N	ATAAAGCTTNNNNNNNNNNNNNNNACTAGTTTTACGGCTAGCTCAG
T1_rev	TACTCAGGAGAGCGTTCACC

Table S9. Primers used to generate barcoded TEVp libraries A, B, and AB. Degenerate codons and barcode regions are highlighted in uppercase. The primer combinations used to generate fragments for the respective libraries are listed in Table S10. The fragments combined to obtain each library are specified in Table S11.

Name	Sequence (5' → 3')
TEVp_PacI	atattaattaattacagctgggtcgcttctttaac
TEVp_BC_12N	ataaagcttNNNNNNNNNNNNactagttttacggctagctcag
TEVp_29	ggtatgaccatcactttcattgg
TEVp_30-32_NNK	ccaatgaaagtgatggtcataccNNKNNKNNKtatggtattggttttggtccg
TEVp_BC_15N	ataaagcttNNNNNNNNNNNNNNNactagttttacggctagctcag
TEVp_217-219_NNS	atattaattaattacagctgggtcgcttctttaaccggctgaaacggttcttccggtttSNNSNNSNNaactttatgaccac cccac
TEVp_216	aactttatgaccaccccac
TEVp_33	tatggtattggttttggtccg

Table S10. Primer combinations used to generate fragments for libraries A, B, and AB. The corresponding primer sequences can be found in Table S9. The fragments combined to obtain each library are specified in Table S11.

PCR fragment	Template	Forward primer	Reverse primer
Fragment 1	Plasmid	TEVp_30-32_NNK	TEVp_PacI
Fragment 2	Plasmid	TEVp_BC_12N	TEVp_29
Fragment 3	Plasmid	TEVp_BC_15N	TEVp_216
Fragment 4	Fragment 3	TEVp_BC_15N	TEVp_217-219_NNS
Fragment 5	Plasmid	TEVp_33	TEVp_216
Fragment 6	Plasmid	TEVp_BC_15N	TEVp_29
Fragment 7	Fragment 5	TEVp_30-32_NNK	TEVp_217-219_NNS

Table S11. Assembly scheme for fragments from Table S10 to generate libraries A, B and AB. Fragments were joined using overlap extension PCR with the indicated primers. Fragment numbers correspond to the numbers in Table S10. Primer sequences can be found in Table S9.

Library	Mutated TEVp residues	Fragments used/joined	Forward primer	Reverse primer
A	30, 31, 32	1+2	TEVp_BC_12N	TEVp_PacI
B	217, 218, 219	4	TEVp_BC_15N	TEVp_PacI
AB	30, 31, 32, 217, 218, 219	6+7	TEVp_BC_15N	TEVp_PacI

Table S12. Primers used to prepare NGS samples for barcode-to-variant assignment for TEVp variants in library A. Annealing regions are highlighted in italics. Flow cell-binding regions are shown in blue. Sequencing primer-binding sites are shown in green. Note that three different forward and reverse primers were used with different staggering elements (underlined) to increase sequence diversity in Illumina cycles.

Primer name	Sequence (5' → 3')
Forward:	
TEVp_30-32_NGSread1	AATGATACGGCGACCACCGAGATCTACACTCTTTCCCTACACGACGCTCTTCCGATCT*TCGGACCAAAACCAATACC*
TEVp_30-32_NGSread2	AATGATACGGCGACCACCGAGATCTACACTCTTTCCCTACACGACGCTCTTCCGATCTTA*CGGACCAAAACCAATACC*
TEVp_30-32_NGSread3	AATGATACGGCGACCACCGAGATCTACACTCTTTCCCTACACGACGCTCTTCCGATCTTAC*CGGACCAAAACCAATACC*
Reverse:	
TEVp_BC_NGSread1	CAAGCAGAAGACGGCATACGAGATGTGACTGGAGTTCAGACGTGTGCTCTTCCGATCTT*CTAATCCTTTCACTGCAG*
TEVp_BC_NGSread2	CAAGCAGAAGACGGCATACGAGATGTGACTGGAGTTCAGACGTGTGCTCTTCCGATCTT*ACTAATCCTTTCACTGCAG*
TEVp_BC_NGSread3	CAAGCAGAAGACGGCATACGAGATGTGACTGGAGTTCAGACGTGTGCTCTTCCGATCTT*ACCTAATCCTTTCACTGCAG*

Table S13. Search space and best values for hyperparameters during optimization of the MLP model.

Hyperparameter	Type	Range (start, end, step)	Best value
Hidden layers (l)	int	1, 5, 1	1
Units per layer (h)	int	50, 1000, 10	440
Dropout rate (p)	float	0, 0.5, 0.1	0.3
Weight power (ρ)	float	0, 2, 0.1	0.8
Focal loss alpha (α)	float	0, 1, 0.1	0.2
Focal loss gamma (γ)	float	0, 3, 0.1	0.8
Learning rate (η)	float	1e-4, 1e-2, 1e-4	7e-4
Embedding (ω)	categorical	OneHot, BLOSUM30-100	BLOSUM100
Read threshold train (θ)	int	0, 500, 10	20
Clip weights (c)	Int	10, 500, 10	260

Table S14. Search space and best values for hyperparameters during optimization of the ESM prediction head.

Hyperparameter	Type	Range (start, end, step)	Best value
Hidden layers (l)	int	1, 5, 1	1
Units per layer (h)	int	50, 1000, 10	640
Dropout rate (p)	float	0, 0.5, 0.1	0.5
Weight power (ρ)	float	0, 2, 0.1	0.5
Focal loss alpha (α)	float	0, 1, 0.1	0.1
Focal loss gamma (γ)	float	0, 3, 0.1	1.5
Learning rate (η)	float	1e-4, 1e-2, 1e-4	7.1e-4
Read threshold train (θ)	int	0, 500, 10	0
Clip weights (c)	Int	10, 500, 10	230

Table S15. Search space and best values for hyperparameters during optimization of the GBT model.

Hyperparameter	Type	Range (start, end, step)	Best value
Loss	categorical	Log, Exponential	Log
Learning rate	float	1e-3, 1e-1, 1e-3	9.3e-2
Estimators	int	1, 100, 1	97
Criterion	categorical	Squared Error, Friedman MSE	Squared Error
Max depth	int	1, 100, 1	50
Min samples split	int	2, 100, 1	65
Min samples leaf	int	1, 100, 1	83
Max leaf nodes	int	1, 100, 1	161
Embedding (ω)	categorical	OneHot, BLOSUM30-100	BLOSUM60
Read threshold train (θ)	int	0, 500, 10	110

Table S16. Search space and best values for hyperparameters during optimization of the SVC model.

Hyperparameter	Type	Range (start, end, step)	Best value
Loss	categorical	Hinge, Squared Hinge	Hinge
Regularization	int	50, 1000, 10	0.1, 10, 0.1
Class weight	categorical	None, Balanced	None
Embedding (ω)	categorical	OneHot, BLOSUM30-100	OneHot
Read threshold train (θ)	int	0, 500, 10	500

Table S17. Search space and best values for hyperparameters during optimization of the LR model.

Hyperparameter	Type	Range (start, end, step)	Best value
Penalty	categorical	L1, L2, ElasticNet	ElasticNet
Regularization	int	50, 1000, 10	0.1, 10, 0.1
Class weight	categorical	None, Balanced	None
Embedding (ω)	categorical	OneHot, BLOSUM30-100	BLOSUM30
Read threshold train (θ)	int	0, 500, 10	470

Table S18. Search space and best values for hyperparameters during optimization of the KNN model.

Hyperparameter	Type	Range (start, end, step)	Best value
Neighbors	int	1, 100, 1	10
Weights	categorical	Uniform, Distance	Distance
Metric	categorical	Chebyshev, Cityblock, Cosine, Euclidean	Cosine
Embedding (ω)	categorical	OneHot, BLOSUM30-100	BLOSUM30
Read threshold train (θ)	int	0, 500, 10	30

Supplementary notes

Supplementary note 1. Sequence of plasmid pProtrec. The plasmid contains a dedicated 12 nt long TEVp BC generated from an N_{12} primer overhang and associated with variant TEVp I (TACTACGAAACA, highlighted in bold) as well as the 6 nt long TEVs BC B001 indicative of ENLYFQS (CGATGT, highlighted in bold). The HindIII site between the TEVp BC and TEVs BC together with PacI were used to insert the TEVp libraries and are highlighted in bold and blue. XhoI located within *sfGFP* and NotI located right after the stop codon downstream of *ssrA* were frequently used for cloning and are highlighted in bold and red. SpeI and NcoI used to generate short fragments for Illumina NGS readout of our recorder are underlined and highlighted in italic. The pSEVA291 backbone from SanDI to PacI is omitted. SanDI and PacI are underlined.

TTAATTAATTACAGCTGGGTCGCTTCTTTAACCGGCTGAAACGGTTCTTCCGGTTTCACCATAAAAACTTTATGACCACCCCA
CAGAACGCTATCTGCATTCAGACGCCAACCGCTAACCCACTGCTGTGCCTCTTGATTGGTCAGCAGTTCCATGAAGTTTTTCG
GCACGCTGGTAAAATAGTTGATGGTATTGGTAAAGTTGCTGGCGCTATGAATACCAACAATAAAACCATCACGGGTGCTAACC
AGCGGACTACCACACTGACCATCTTTGGTCTGAATCCAATGTTTCCAAAAAATACCATCACCGCTCGGAAAGGTACAGCTGGT
ATCGCTAACCATGCTGCTCATTCTTTTGGTCTGAAAATTGGTGGTAACCAGACAAATACGTTCTTCACGCTGCGGTTCACGAA
ATTTCAGTTTCTGCGGAAAAGGCGGAAAATCTTTCGGCATACGAATAATAATCATATCACGACCATCAACCAGATGCTGCTGC
AGGGTGGTGGTATCTTTAACTTTAAAAACACCATGCAGGCTCTGAACCACCAGGGTGCCATTATTGCGACGAAACAGGTGTTT
ATTGGTGATAATAAACGGACCAAAACCAATACCATACAGACTGGTGGTATGACCATCACTTTCATTGGTCAGATGGCAAATGC
TGCTGCTAATCGGATTATAATCACGCGGACCTTTAAACAGGCTTTCGCCCATATGTATATCTCCTTCTTAAAGTTAAACAAAA
TTATTTCTAGACCGGTGCTAGCATAATACCTAGGGCTGAGCTAGCCGTAAA*ACTAGT***TACTACGAAACAAAGCTTCGATGT**CT
GCAGTGAAAGGATTAGGGGAACGCAGAGCGTGTAGAATTTCGTTAAACGTTGGGGTTTATCGTCTCTAGTTATTATCCACGAG
ACGCGGGTACGGCGGGGCGGTCACGACAGACTCTTGGGGCTAATCGACCAGAGCTTCGGGTAAGAGTTGATACAAACAATCCC
TCAAAGATCGGGCCCTAAAGCTCGCAAGATTTAACCACTCGGCCGGCTTGTCGACGACGGCGGTCTCCGTCGTCAGGATCATC
CGGGCATCGCTTAGTCACCTTTGGGCCACGGTCCGCTACCTTACAGGAATAGTACTCGTCCTTTAATTTGGAATGAA*CCATGG*
CAGTCAGTTGTGTTGCGTTTCTTCGACCTAGTACTCGCTCCCTTAGGAGAAAGACAGATAGCTTCTTACCCGGGGTTTGTACC
GTACACCACTGAGACCGCGGTGGTTGACCAGACAAACCACGAAGGTTCTGTTAAGTAACTGAACCCAATGTCGTTAGTGACGC
TTACCTCTTAAGAGGTCACTGACCTAACAGGATCCCACCACAATTCAGCAAATTGTGAACATCATCACGTTCATCTTTCCCTG
GTTGCCAATGGCCCATTTTCCTGTCAGTAACGAGAAGGTCGCGAATTCAGGCGCTTTTTAGACTGGTCGTAATGAAGGGTACC
ATAAATGCTTCAATAATACTCTGGATGTAATGTGATGCGAGCTCTGGTTGTTATTCGTCTGAGCCGTGTTACCGATGCAACCA
CCAGTCCGGAACGTCAGCTGGAAAGCTGTCAGCAGCTGTGTGCACAGCGTGGTTGGGATGTTGTTGGTGTTGCAGAGGATCTG
GATGTTAGCGGTGCAGTTGATCCGTTTGATCGTAAACGTCGTCCGAATCTGGCACGTTGGCTGGCATTTGAAGAACAGCCGTT
TGATGTTATTGTTGCCTATCGTGTTGATCGTCTGACCCGTAGCATTCGTCATCTGCAACAGCTGGTTCATTGGGCAGAAGATC
ATAAAAAACTGGTTGTGAGCGCAACCGAAGCACATTTTGATACCACCACCCCGTTTGCAGCAGTTGTTATTGCACTGATGGGC
ACCGTTGCACAGATGGAACTGGAAGCAATTAAAGAACGTAATCGTAGCGCAGCCCATTTTAACATTCGTGCAGGTAAATATCG
TGGTAGCCTGCCTCCGTGGGGTTATCTGCCGACGCGTGTAGATGGTGAATGGCGTCTGGTTCCTGACCCGGTTCAGCGTGAAC
GTATTCTGGAAGTATATCATCGTGTGGTGGATAATCATGAACCGCTGCATCTGGTTGCACATGATCTGAATCGTCGTGGTGTT
CTGAGTCCCAAAGATTATTTTGCTCAGCTGCAAGGTCGTGAACCGCAGGGTCGTGAATGGTCTGCAACCGCACTGAAACGTAG
CATGATTAGCGAAGCAATGCTGGGTTATGCAACCCTGAATGGTAAAACCGTTCGTGATGATGATGGTGCACCGCTGGTTCGTG
CAGAACCGATTCTGACACGTGAACAGCTGGAAGCACTGCGTGCCGAACTGGTTAAAACCAGCCGTGCAAAACCGGCAGTTAGC
ACCCCGAGCCTGCTGCTGCGTGTTCTGTTTTGTGCAGTTTGTGGTGAACCGGCATACAAATTTGCCGGTGGTGGTCGTAAACA
TCCGCGTTATCGTTGTCGTAGCATGGGTTTTCCGAAACATTGTGGTAATGGTACAGTTGCAATGGCAGAATGGGATGCATTTT
GCGAAGAACAGGTTCTGGATCTGCTGGGTGATGCCGAACGTCTGGAAAAAGTTTGGGTTGCAGGTAGCGATAGCGCAGTTGAA
CTGGCCGAAGTTAATGCGGAACTGGTCGATCTCACCAGTCTGATTGGAAGTCCCGCATATCGTGCGGGTAGTCCTCAGCGTGA
AGCACTGGATGCACGTATTGCAGCACTGGCAGCACGTCAAGAAGAACTGGAAGGTCTGGAAGCACGTCCGAGCGGTTGGGAAT
GGCGTGAAACAGGTCAGCGTTTTGGTGATTGGTGGCGTGAGCAGGATACCGCAGCAAAAAATACCTGGCTGCGTAGTATGAAT
GTTCGCCTGACCTTTGATGTTCGCGGTGGCCTGACCCGCACCATTGATTTTGGCGATCTGCAAGAATATGAACAGCATCTGCG
TCTGGGTAGCGTTGTTGAACGTCTGCATACCGGCATGAGCACCGGCGGTGGCAGCGGCGGTTCTGGTGGCTCTAGCAAAGGAG
AAGAACTTTTCACTGGAGTTGTCCCAATTCTTGTTGAATTAGATGGTGATGTTAATGGGCACAAATTTTCTGTCCGTGGAGAG
GGTGAAGGTGATGCTACAAACGGAAAACTCACCCTTAAATTTATTTGCACTACTGGAAAACTACCTGTTCCGTGGCCAACACT
TGTCACTACTCTGACCTATGGTGTTCAATGCTTTTCCCGTTATCCGGATCACATGAAACGGCATGACTTTTTCAAGAGTGCCA
TGCCCGAAGGTTATGTACAGGAACGCACTATATCTTTCAAAGATGACGGGACCTACAAGACGCGTGCTGAAGTCAAGTTTGAA
GGTGATACCCTTGTTAATCGTATCGAGTTAAAGGGTATTGATTTTAAAGAAGATGGAAACATTCTTGGACACAAA**CTCGAG**TA
CAACTTTAACTCACACAATGTATACATCACGGCAGACAAACAAAAGAATGGAATCAAAGCTAACTTCAAAATTCGCCACAACG
TTGAAGATGGTTCCGTTCAACTAGCAGACCATTATCAACAAAATACTCCAATTGGCGATGGCCCTGTCCTTTTACCAGACAAC
CATTACCTGTCGACACAATCTGTCCTTTCGAAAGATCCCAACGAAAAGCGTGACCACATGGTCCTTCTTGAGTTTGTAACTGC
TGCTGGGATTACACATGGCATGGATGAACTCTACAAAAGGCCTGGTAGTGCTGGCTCCGCTGCTGGTTCTGGCTCGGGCGAAA
ACCTGTATTTTCAGAGCGCTAGCGGTGGCAGCGGAGGTTCGGGGGGCAGTGCGGCGAACGATGAAAACTATAACTATGCGCTG
GCGGCGTAATAA**GCGGCCGC**GTCGTGACTGGGAAAACCCTGGCGCTAGTCTTGGACTCCTGTTGATAGATCCAGTAATGACCT
CAGAACTCCATCTGGATTTGTTCAGAACGCTCGGTTGCCGCCGGGCGTTTTTTATTGGTGAGAATCCAGGGGTCCC

Supplementary note 2. Sequence of the pProtRec derivative lacking the *ssrA* tag (positive control, max. signal). This plasmid was used as a positive control to mimic full proteolytic cleavage by TEVp, which corresponds to a quantitative removal of the SsrA-tag. A unique barcode was added to facilitate identification during NGS. Shown is only the region from *Xho*I (within *sfGFP*) and *Not*I (restriction sites bold underlined). The remaining sequence is identical to pProtRec.

CTCGAGTACAACTTTAACTCACACAATGTATACATCACGGCAGACAAACAAAAGAATGGAATCAAAGCTAACTTCAAAATTCG
CCACAACGTTGAAGATGGTTCCGTTCAACTAGCAGACCATTATCAACAAAATACTCCAATTGGCGATGGCCCTGTCCTTTTAC
CAGACAACCATTACCTGTCGACACAATCTGTCCTTTCGAAAGATCCCAACGAAAAGCGTGACCACATGGTCCTTCTTGAGTTT
GTAACTGCTGCTGGGATTACACATGGCATGGATGAACTCTACAAAAGGCCTGGTAGTGCTGGCTCCGCTGCTGGTTCTGGCTC
GGGCGAAAACCTGTATTTTCAGTAATAA**GCGGCCGC**

Supplementary note 3. Sequence of the pProtRec derivative lacking the promoter and CDS of TEVp (negative control). This plasmid lacks both promoter and CDS of TEVp. A unique barcode was added to facilitate identification during NGS. Shown is only the region from *Pac*I to *Hind*III (bold underlined). The remaining sequence is identical to pProtRec.

TTAATTAAAGCGGATAACAATTTCACACAGGAACTAGTATTTGCATTGAT**AAGCTT**

Supplementary note 4. Sequence of the pProtRec derivative coding for mCherry instead of a functional TEVp (negative control, min. signal). This plasmid was used as a negative control and to mimic the absence of proteolytic cleavage by TEVp. For this, the CDS of TEVp I was replace by a fusion of the first 13 codons of TEVp (to ensure comparable expression levels to TEVp variants) followed by the CDS of mCherry. A unique barcode was added to facilitate identification during NGS. Shown is only the region from *Pac*I to *Hind*III (bold underlined). The remaining sequence is identical to pProtRec.

TTAATTAATTAAGGCCTCTTGTACAGCTCGTCCATGCCGCCGGTGGAGTGGCGGCCCTCGGCGCGTTCGTACTGTTCCACGAT
GGTGTAGTCCTCGTTGTGGGAGGTGATGTCCAACTTGATGTTGACGTTGTAGGCGCCGGGCAGCTGCACGGGCTTCTTGGCCT
TGTAGGTGGTCTTGACCTCAGCGTCGTAGTGGCCGCCGTCCTTCAGCTTCAGCCTCTGCTTGATCTCGCCCTTCAGGGCGCCG
TCCTCGGGGTACATCCGCTCGGAGGAGGCCTCCCAGCCCATGGTCTTCTTCTGCATTACGGGGCCGTCGGAGGGGAAGTTGGT
GCCGCGCAGCTTCACCTTGTAGATGAACTCGCCGTCCTGCAAGGAGGAGTCCTGGGTCACGGTCACCACGCCGCCGTCCTCGA
AGTTCATCACGCGCTCCCACTTGAAGCCCTCGGGGAAGGACAGCTTCAAGTAGTCGGGGATGTCGGCGGGGTGCTTCACGTAG
GCCTTGGAGCCGTACATGAACTGAGGGGACAGGATGTCCCAGGCGAAGGGCAGGGGGCCACCCTTGGTCACCTTCAGCTTGGC
GGTCTGGGTGCCCTCGTAGGGGCGGCCCTCGCCCTCGCCCTCGATCTCGAACTCGTGGCCGTTCACGGAGCCCTCCATGTGCA
CCTTGAAGCGCATGAACTCCTTGATGATGGCCATGTTATCTTCTTCGCCCTTGCTATGCGGATTATAATCACGCAGACCTTTA
AACAGGCTTTCGCCCATATGTATATCTCCTTCTTAAAGTTAAACAAAATTATTTCTAGACCGGTGCTAGCATAATACCTAGGG
CTGAGCTAGCCGTAAAACTAGTCTCCTTTAATAC**AAGCTT**

Supplementary note 5. Sequence of the pProtRec derivative used to facilitate cloning of TEVp libraries. The plasmid harbors a large "mock" insert (fusion protein of maltose binding protein, mCherry and TEVp 0 C151A) in place of the functional TEVp I CDS. Shown is only the region between *Pac*I and *Hind*III sites (bold underlined). The remaining sequence is identical to pProtRec.

TTAATTAATTATTACAGCTGGGTCGCTTCTTTAACCGGCTGAAACGGTTCTTCCGGTTTCACCATAAAAACTTTATGACCACC
CCACAGAACGCTATCTGCATTCAGACGCCAACCGCTAACCCACTGCTGTGCCTCTTGATTGGTCAGCAGTTCCATGAAGTTTT
TCGGCACGCTGGTAAAATAGTTGTTGGTATTGGTAAAGTTGCTGGCGCTATGAATACCAACAATAAAACCATCACGGGTGCTA
ACCAGCGGACTACCCGCCTGACCATCTTTGGTCTGAATCCAATGTTTCCAAAAAATACCATCACCGCTCGGAAAGGTACAGCT
GGTATCGCTAACCATGCTGCTCATGCTTTTGGTCTGAAAATTGGTGGTAACCAGACAAATACGTTCTTCACGCTGCGGTTCAC
GAAATTTCAGTTTCTGCGGAAAAGGCGGAAAATCTTTCGGCATACGAATAATAATCATATCACGACCATCAACCAGATGCTGC
TGCAGGGTGGTGGTATCTTTAACTTTAAAAACACCATGCAGGCTCTGAACCACCAGGGTGCCATTATTGCGACGAAACAGGTG
TTTATTGGTGATAATAAACGGACCAAAACCAATACCATACAGACTGGTGGTATGACCATCACTTTCATTGGTCAGATGGCAAA
TGCTGCTGCTAATCGGATTATAATCACGCGGACCTTTAAACAGGCTTTCGCCGGTACCGCCAGAACCAGCAGCGGAGCCAGCG
GATCCAGGCCTCTTGTACAGCTCGTCCATGCCGCCGGTGGAGTGGCGGCCCTCGGCGCGTTCGTACTGTTCCACGATGGTGTA
GTCCTCGTTGTGGGAGGTGATGTCCAACTTGATGTTGACGTTGTAGGCGCCGGGCAGCTGCACGGGCTTCTTGGCCTTGTAGG
TGGTCTTGACCTCAGCGTCGTAGTGGCCGCCGTCCTTCAGCTTCAGCCTCTGCTTGATCTCGCCCTTCAGGGCGCCGTCCTCG
GGGTACATCCGCTCGGAGGAGGCCTCCCAGCCCATGGTCTTCTTCTGCATTACGGGGCCGTCGGAGGGGAAGTTGGTGCCGCG
CAGCTTCACCTTGTAGATGAACTCGCCGTCCTGCAAGGAGGAGTCCTGGGTCACGGTCACCACGCCGCCGTCCTCGAAGTTCA
TCACGCGCTCCCACTTGAAGCCCTCGGGGAAGGACAGCTTCAAGTAGTCGGGGATGTCGGCGGGGTGCTTCACGTAGGCCTTG
GAGCCGTACATGAACTGAGGGGACAGGATGTCCCAGGCGAAGGGCAGGGGGCCACCCTTGGTCACCTTCAGCTTGGCGGTCTG
GGTGCCCTCGTAGGGGCGGCCCTCGCCCTCGCCCTCGATCTCGAACTCGTGGCCGTTCACGGAGCCCTCCATGTGCACCTTGA
AGCGCATGAACTCCTTGATGATGGCCATGTTATCTTCTTCGCCCTTGCTATGGCCGCCGCCGGAGCCAGTCTGCGCGTCTTTC
AGGGCTTCATCGACAGTCTGACGACCGCTGGCGGCGTTGATCACCGCAGTACGCACGGCATACCAGAAAGCGGACATCTGCGG
GATGTTCGGCATGATTTCACCTTTCTGGGCGTTTTCCATGGTGGCGGCAATACGTGGATCTTTCGCCAACTCTTCCTCGTAAG
ACTTCAGCGCTACGGCACCCAGCGGTTTGTCTTTATTAACCGCTTCCAGACCTTCATCAGTCAGCAGATAGTTTTCGAGGAAC
TCTTTCGCCAGCTCTTTGTTCGGACTGGCGGCGTTAATACCTGCGCTCAGCACGCCAACGAACGGTTTGGATGGTTGACCCTT
GAAGGTCGGCAGTACCGTTACACCATAATTCACTTTGCTGGTGTCGATGTTGGACCATGCCCACGGGCCGTTGATGGTCATCG
CTGTTTCGCCTTTATTAAAGGCAGCTTCTGCGATGGAGTAATCGGTGTCTGCATTCATGTGTTTGTTTTTAATCAGGTCAACC
AGGAAGGTCAGACCCGCTTTCGCGCCAGCGTTATCCACGCCCACGTCTTTAATGTCGTACTTGCCGTTTTCATACTTGAACGC
ATAACCCCCGTCAGCAGCAATCAGCGGCCAGGTGAAGTACGGTTCTTGCAGGTTGAACATCAGCGCGCTCTTACCTTTCGCTT
TCAGTTCTTTATCCAGCGCCGGGATCTCTTCCCAGGTTTTTGGCGGGTTCGGCAGCAGATCTTTGTTATAAATCAGCGATAAC
GCTTCAACAGCGATCGGGTAAGCAATCAGCTTGCCGTTGTAACGTACGGCATCCCAGGTAAACGGATACAGCTTGTCCTGGAA
CGCTTTGTCCGGGGTGATTTCAGCCAACAGGCCAGATTGAGCGTAGCCACCAAAGCGGTCGTGTGCCCAGAAGATAATGTCAG
GGCCATCGCCAGTTGCCGCAACCTGTGGGAATTTCTCTTCCAGTTTATCCGGATGCTCAACGGTGACTTTAATTCCGGTATCT
TTCTCGAATTTCTTACCGACTTCAGCGAGACCGTTATAGCCTTTATCGCCGTTAATCCAGATTACCAGTTTACCTTCTTCAGT
TTTCATATGTATATCTCCTTCTTAAAGTTAAACAAAATTATTTCTAGACCGGTGCTAGCATAATACCTAGGGCTGAGCTAGCC
GTAAAACTAGTAGCAGCCTACCG**AAGCTT**

Supplementary note 6. Sequence of the pProtRec derivative used to facilitate cloning of the TEVs single-site variant library. The plasmid is based on pProtRec but harbors an *Sph*I site (bold) downstream of *tevs* to facilitate library cloning. Shown is only the region from *Xho*I (located within *sfGFP*) to *Not*I (restriction sites bold underlined) located downstream of *ssrA*. To generate pProtrec3, an SphI restriction site was inserted next to TEVs.

CTCGAGTACAACTTTAACTCACACAATGTATACATCACGGCAGACAAACAAAAGAATGGAATCAAAGCTAACTTCAAAATTCG
CCACAACGTTGAAGATGGTTCCGTTCAACTAGCAGACCATTATCAACAAAATACTCCAATTGGCGATGGCCCTGTCCTTTTAC
CAGACAACCATTACCTGTCGACACAATCTGTCCTTTCGAAAGATCCCAACGAAAAGCGTGACCACATGGTCCTTCTTGAGTTT
GTAACTGCTGCTGGGATTACACATGGCATGGATGAACTCTACAAAAGGCCTGGTAGTGCTGGCTCCGCTGCTGGTTCTGGCTC
GGGCGAAAACCTGTATTTTCAGAGCGCTAGC**GCATGC**GGTGGCAGCGGAGGTTCGGGGGGCAGTGCGGCGAACGATGAAAACT
ATAACTATGCGCTGGCGGCGTAATAA**GCGGCCGC**

Supplementary note 7. Sequence of the pProtRec derivative lacking both linkers adjacent to *tevs*. Shown is only the region from *Xho*I (within *sfGFP*) to *Not*I (restriction sites bold underlined).

CTCGAGTACAACTTTAACTCACACAATGTATACATCACGGCAGACAAACAAAAGAATGGAATCAAAGCTAACTTCAAAATTCG
CCACAACGTTGAAGATGGTTCCGTTCAACTAGCAGACCATTATCAACAAAATACTCCAATTGGCGATGGCCCTGTCCTTTTAC
CAGACAACCATTACCTGTCGACACAATCTGTCCTTTCGAAAGATCCCAACGAAAAGCGTGACCACATGGTCCTTCTTGAGTTT
GTAACTGCTGCTGGGATTACACATGGCATGGATGAACTCTACAAAAGGCCTGCTAGCGAAAACCTGTATTTTCAGAGCGCTAG
CGCGGCGAACGATGAAAACTATAACTATGCGCTGGCGGCGTAATAA**GCGGCCGC**

Supplementary note 8. Sequence of the pProtRec derivative lacking the linker downstream of *tevs*. Shown is only the region from *Xho*I (within *sfGFP*) to *Not*I (restriction sites bold underlined).

CTCGAGTACAACTTTAACTCACACAATGTATACATCACGGCAGACAAACAAAAGAATGGAATCAAAGCTAACTTCAAAATTCG
CCACAACGTTGAAGATGGTTCCGTTCAACTAGCAGACCATTATCAACAAAATACTCCAATTGGCGATGGCCCTGTCCTTTTAC
CAGACAACCATTACCTGTCGACACAATCTGTCCTTTCGAAAGATCCCAACGAAAAGCGTGACCACATGGTCCTTCTTGAGTTT
GTAACTGCTGCTGGGATTACACATGGCATGGATGAACTCTACAAAAGGCCTGGTAGTGCTGGCTCCGCTGCTGGTTCTGGCTC
GGGCGAAAACCTGTATTTTCAGAGCGCTAGCGCGGCGAACGATGAAAACTATAACTATGCGCTGGCGGCGTAATAA**GCGGCCG**
C

Supplementary note 9. Sequence of the pProtRec region containing *tevs* flanked by two adjacent linkers. Shown is only the region from *Xho*I (within *sfGFP*) to *Not*I (restriction sites bold underlined).

CTCGAGTACAACTTTAACTCACACAATGTATACATCACGGCAGACAAACAAAAGAATGGAATCAAAGCTAACTTCAAAATTCG
CCACAACGTTGAAGATGGTTCCGTTCAACTAGCAGACCATTATCAACAAAATACTCCAATTGGCGATGGCCCTGTCCTTTTAC
CAGACAACCATTACCTGTCGACACAATCTGTCCTTTCGAAAGATCCCAACGAAAAGCGTGACCACATGGTCCTTCTTGAGTTT
GTAACTGCTGCTGGGATTACACATGGCATGGATGAACTCTACAAAAGGCCTGGTAGTGCTGGCTCCGCTGCTGGTTCTGGCTC
GGGCGAAAACCTGTATTTTCAGAGCGCTAGCGGTGGCAGCGGAGGTTCGGGGGGCAGTGCGGCGAACGATGAAAACTATAACT
ATGCGCTGGCGGCGTAATAA**GCGGCCGC**

Supplementary note 10. Parent sequence of the TEVp single-site variant library obtained as synthetic DNA fragment pools. The CDS of the parent TEVp 0 is highlighted in upper-case with start and stop codon underlined.

actagttttacggctagctcagccctaggtattatgctagcaccggtctagaaataattttgtttaactttaagaaggagata
tacatATGGGCGAAAGCCTGTTTAAAGGTCCGCGTGATTATAATCCGATTAGCAGCAGCATTTGCCATCTGACCAATGAAAGT
GATGGTCATACCACCAGTCTGTATGGTATTGGTTTTGGTCCGTTTATTATCACCAATAAACACCTGTTTCGTCGCAATAATGG
CACCCTGGTGGTTCAGAGCCTGCATGGTGTTTTTAAAGTTAAAGATACCACCACCCTGCAGCAGCATCTGGTTGATGGTCGTG
ATATGATTATTATTCGTATGCCGAAAGATTTTCCGCCTTTTCCGCAGAAACTGAAATTTCGTGAACCGCAGCGTGAAGAACGT
ATTTGTCTGGTTACCACCAATTTTCAGACCAAAAGCATGAGCAGCATGGTTAGCGATACCAGCTGTACCTTTCCGAGCGGTGA
TGGTATTTTTTGGAAACATTGGATTCAGACCAAAGATGGTCAGTGTGGTAGTCCGCTGGTTAGCACCCGTGATGGTTTTATTG
TTGGTATTCATAGCGCCAGCAACTTTACCAATACCAACAACTATTTTACCAGCGTGCCGAAAAACTTCATGGAACTGCTGACC
AATCAAGAGGCACAGCAGTGGGTTAGCGGTTGGCGTCTGAATGCAGATAGCGTTCTGTGGGGTGGTCATAAAGTTTTTATGGT
GAAACCGGAAGAACCGTTTCAGCCGGTTAAAGAAGCGACCCAGCTGTAAttaattaaaggcatcaaataaaacgaaaggctca
gtcgaaagactgggcctttcgttttatctgttgtttgtcggtgaacgctctcctgagta

Supplementary References

1. Pestalozzi, L. M. Directed evolution of tobacco etch virus protease towards higher in vitro activity. (ETH Zurich, 2019). https://www.research-collection.ethz.ch/bitstream/handle/20.500.11850/370837/4/PhDthesis_LP_no_CV.pdf

2. Höllerer, S. *et al.* Large-scale DNA-based phenotypic recording and deep learning enable highly accurate sequence-function mapping. *Nat. Commun.* **11**, 1–15 (2020).

3. iGEM. Anderson Collection of Promoters. http://parts.igem.org/Promoters/Catalog/Anderson (2006).

4. Olson, C. A., Wu, N. C. & Sun, R. A Comprehensive Biophysical Description of Pairwise Epistasis throughout an Entire Protein Domain. *Curr. Biol.* **24**, 2643–2651 (2014).

5. Phan, J. *et al.* Structural basis for the substrate specificity of tobacco etch virus protease. *J. Biol. Chem.* **277**, 50564–50572 (2002).

6. Kapust, R. B., Tözsér, J., Copeland, T. D. & Waugh, D. S. The P1' specificity of tobacco etch virus protease. *Biochem. Biophys. Res. Commun.* **294**, 949–955 (2002).

7. Martínez-García, E., Aparicio, T., Goñi-Moreno, A., Fraile, S. & de Lorenzo, V. SEVA 2.0: an update of the Standard European Vector Architecture for de-/re-construction of bacterial functionalities. *Nucleic Acids Res.* **43**, D1183–D1189 (2015).

8. Höllerer, S. & Jeschek, M. Ultradeep characterisation of translational sequence determinants refutes rare-codon hypothesis and unveils quadruplet base pairing of initiator tRNA and transcript. *Nucleic Acids Res.* **51**, 2377–2396 (2023).
